# Technologies for the diagnosis of angle closure glaucoma (ACE): protocol of a prospective, multicentre, cross-sectional diagnostic study

**DOI:** 10.1136/bmjopen-2023-073975

**Published:** 2023-10-04

**Authors:** Augusto Azuara-Blanco, John G Lawrenson, Anthony J King, Paul J Foster, Gianni Virgili, Mary Guiney, Winnie Nolan, Anish Jindal, Meenakshi Sharma, Ciaran O'Neill, Christopher R Cardwell, Mike Clarke

**Affiliations:** 1 Centre for Public Health, Queen's University Belfast, Belfast, UK; 2 Department of Optometry and Visual Sciences, City University of London, London, UK; 3 Department of Ophthalmology, Nottingham University Hospitals NHS Trust, Nottingham, UK; 4 Institute of Ophthalmology, Division of of Epidemiology, University College London, London, UK; 5 Northern Ireland Clinical Trials Unit, Belfast Health and Social Care Trust, Belfast, UK; 6 NIHR Biomedical Research Centre, Moorfields and UCL Institute of Ophthalmology, London, UK; 7 Glaucoma Service, Moorfields and Institute of Ophthalmology, Faculty of Brain Sciences, University College London, London, UK; 8 Institute of Ophthalmology, Faculty of Brain Sciences, University College London, London, UK; 9 Northern Ireland Methodology Hub, Centre for Public Health, Queen's University Belfast, Belfast, UK

**Keywords:** ophthalmology, glaucoma, clinical trial, diagnostic imaging

## Abstract

**Introduction:**

Angle-closure is responsible for half of all glaucoma blindness globally. Patients with suspected glaucoma require assessment of the drainage angle by an experienced clinician. The goal of this study is to evaluate the diagnostic performance and cost-effectiveness of two non-contact tests, anterior segment OCT (Optical Coherence Tomography) (AS-OCT) and limbal anterior chamber depth for patients referred to hospital with suspected angle closure compared with gonioscopy by ophthalmologist.

**Methods and analysis:**

Study design: prospective, multicentre, cross-sectional diagnostic accuracy study. Inclusion criteria: adults referred from community optometry to hospital with suspected angle closure. Primary outcome: Sensitivity and specificity. Secondary outcomes: Positive/negative likelihood ratios, concordance, cost-effectiveness, proportion of patients requiring subsequent clinical assessment by ophthalmologist. Sample size: 600 individuals who have been referred with suspected angle closure from primary care (community optometry). We will have a 95% probability of detecting the true sensitivity of either test to within ±3.5% based on a sensitivity of 90%. The study would also have a 95% probability of detecting the true specificity of either test to within ±5%, assuming a specificity of 75%.

**Ethics and dissemination:**

Ethical Review Board approval was obtained. REC reference: 22/LO/0885. Our findings will be disseminated to those involved in eye care services. We will have a knowledge exchange event at the end of the study, published via the Health Technology Assessment web page and in specialist journals. The results will be presented at professional conferences and directly to patients via patient group meetings and the Glaucoma UK charity.

**Trial registration number:**

ISRCTN15115867.

**Strengths and limitations of this study**

- Prospective study design.
- Masked investigators interpreting tests and the reference standard.
- Adequate, large sample size.
- Evaluation of cost-effectiveness of the novel patient pathway.
- Imperfect, subjective reference standard.

## Introduction

Glaucoma is a chronic optic neuropathy and a leading cause of irreversible blindness.^1–4^ In the UK, more than 500 000 people have glaucoma, and 4000 new patients are registered each year with sight loss because of glaucoma. Many more people have glaucoma not severe enough to be registered, but severe enough to reduce vision and quality of life (eg, loss of their driving licence).^5^

Hospital eye services (HES) account for 8% of outpatient attendances to the National Health Service (NHS) and glaucoma accounts for 25% of outpatient activity of HES, with more than 1 million visits per year in England alone due to glaucoma.^5^

There are two types of glaucoma, according to the appearance of the drainage angle of the eye: open angle glaucoma (OAG), the most common, and angle closure glaucoma (ACG). In ACG, which is the subject of this study, the drainage angle is blocked, leading to acute or chronic elevation of intraocular pressure and damage of the optic nerve.

ACG is less common but more severe than OAG and its prevalence increases with age (Bourne, Tham). Acute angle closure is an uncommon but serious ocular emergency.^6 7^ In the early stage of the disease, angle closure can be treated with laser to reduce the risk of developing glaucoma and vision loss.^6 8^

ACG is responsible for approximately one in six glaucomas in the UK and a substantial proportion of glaucoma referrals to HES.^9 10^

A recent Cochrane review found suboptimal quality evidence regarding the diagnostic performance of non-contact tests, anterior segment OCT (AS-OCT) and limbal anterior chamber depth (LACD).^11^ Pooled data showed that LACD had high sensitivity and a possibly sufficient specificity for case finding and performed as well as AS-OCT but the authors highlighted ‘There is still a need for high-quality studies to evaluate the performance of non-contact tests for angle assessment’. Given the demands OAG presents the health service and the implications delays in diagnosis may have for patients, clearly it is important to assess the cost-effectiveness of potential improvements to the diagnostic pathway. Our study will fill this gap and determine the potential of these tests in the diagnosis of angle closure.

Our hypothesis is that the non-contact tests for diagnosing angle closure will be accurate and will facilitate a safe and efficient pathway for patients with this condition. The goal of this study is to evaluate the diagnostic performance and cost-effectiveness of two non-contact tests, AS-OCT and LACD for patients referred to hospital with suspected angle closure compared with standard practice (gonioscopy by ophthalmologist). This report describes the protocol V.1.0.

## Methods and analysis

### Study design

ACE is a prospective, cross-sectional, multicentre, diagnostic accuracy study of people referred to HES with suspected angle closure. Study setting is secondary care, HES.

**Figure 1:**
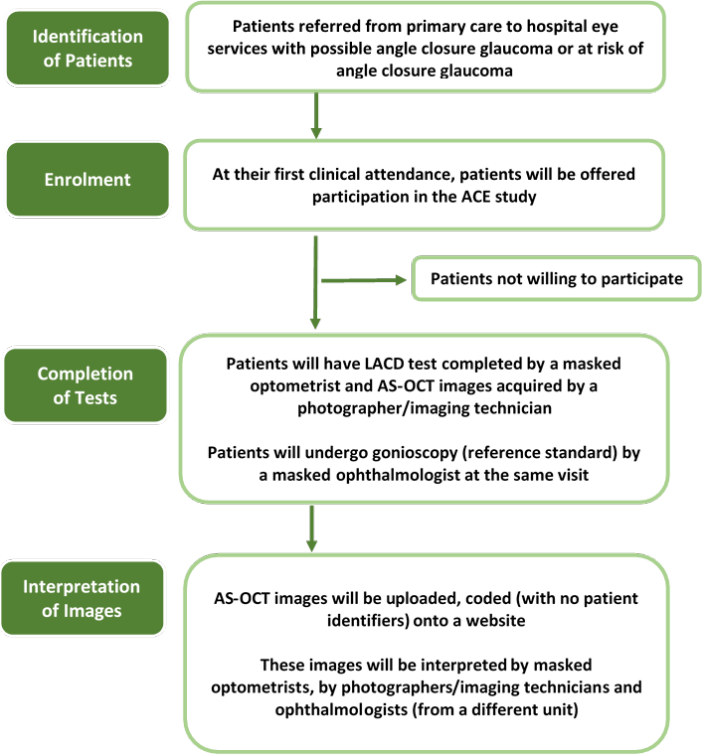
ACE study participant flowchart. AS-OCT, anterior segment Optical Coherence Tomography; LACD, limbal anterior chamber depth.

Sampling and data collection will be carried out prospectively. Consecutive eligible patients will be approached to take part in the study (figure 1). Patients referred to HES with suspected angle closure will be approached and those who consent will undergo testing with the two non-contact technologies (index tests) on the same day. Patients will undergo AS-OCT (with any device available at the site) and the images of the temporal and nasal angle will be sent to a reading centre for interpretation. Interpretation of AS-OCT will use reference images and the investigator will determine if the angle is closed (ie, contact between peripheral iris and trabecular meshwork at one or both quadrants), open or indeterminate. In addition the investigator will grade the angle opening (<10 degrees, 10–20 degrees or >degrees) using reference images. The LACD will be performed and graded by a hospital optometrist (masked to the AS-OCT and to the reference standard). The investigator will use reference images to determine if the angle is closed (ie, contact between peripheral iris and cornea), open or indeterminate. The investigator will also grade the angle opening according to a 7-point scale proposed by Foster *et al*, using reference images.^12^ Patients will then receive the reference standard (gonioscopy), provided by an expert who will be masked to the evaluated tests (LACD and AS-OCT). The expert will be an ophthalmologist with glaucoma expertise and will judge if the angle is open or closed. Masked ophthalmologists, optometrists and trained photographers/imaging technicians will interpret AS-OCTs images provided by the reading centre at a different time.

Prior to the study recruitment we will meet clinicians and investigators at each study site to review and agree on the reference standard and interpretation of tests.

#### Inclusion criteria

Adults (≥18 years) referred from community optometry to HES with suspected angle closure, with or without additional comorbidity.

#### Exclusion criteria

Unable to provide informed consent; unable to undergo a slit-lamp examination.

The primary outcome is sensitivity and specificity of the non-contact tests to detect angle-closure. Reference standard is gonioscopy by an ophthalmologist with glaucoma expertise.

#### Secondary outcomes

Positive/negative likelihood ratios, concordance, cost-effectiveness, proportion of patients requiring subsequent clinical assessment by ophthalmologist, proportion of patients unable to undergo tests and of tests of inadequate quality.

#### Recruitment of study participants

Potential patients will be identified through referral letters from optometrists to HES. Potential patients with possible angle closure will be approached before they come for their routine clinical appointment, by phone or via an invitation letter.

Prior to the initiation of the study, training will be provided to the investigators on the interpretation and grading of the tests (AS-OCT, LACD, gonioscopy) via a web-based teaching module on AS-OCT images and LACD interpretation.

The optometrists performing the LACD test will be masked to gonioscopy. Optometrists, ophthalmic photographers/imaging technicians and ophthalmologists interpreting patients’ images will be masked to the reference standard.

Ophthalmologists performing the gonioscopic evaluation will also be masked to the optometrist LACD test result and to the AS-OCT test, including the findings/decisions made by the optometrists, ophthalmic photographers/imaging technicians and ophthalmologists (who will be reviewing the images at a later date). At the same visit participants will be asked to complete a health status questionnaire, European Quality of Life - five dimension - five level (EQ-5D-5L).^13^ The schedule of assessments is described in online supplemental appendix 1.

10.1136/bmjopen-2023-073975.supp1 Supplementary data

#### Data collection and management

The chief investigator (CI) and the Northern Ireland Clinical Trials Unit (NICTU) will provide training to site staff on trial processes and procedures, including the completion of the case report form (CRF) and data collection. Monitoring during the trial will check the accuracy of entries on CRF’s against the source documents, the adherence to the protocol, procedures and Good Clinical Practice (GCP), as outlined in the trial monitoring plan.

Quality control is implemented by the NICTU in the form of standard operating procedures, which are defined to encompass aspects of the clinical data management process and to ensure standardisation and adherence to International Conference of Harmonisation GCP guidelines and regulatory requirements.

Data validation will be implemented and discrepancy reports will be generated following data entry to identify discrepancies such as out of range, inconsistencies or protocol deviations based on data validation checks programmed in the clinical trial database.

To ensure accurate, complete and reliable data are collected, the NICTU will provide training to site staff either through investigator meetings or site initiation visits.

All data for an individual patient will be collected by the principal investigator (PI) or designee and recorded in source documents and/or the CRF for the study. The EQ-5D-5L questionnaires will be completed by the patient. Patient identification on the CRF and questionnaires will be through their unique trial identifier, allocated at the time of recruitment.

CRFs and questionnaires are to be submitted to the NICTU as per the CRF submission schedule.

In addition to the data specified in the sections above, the following information will be obtained and recorded in the appropriate CRF:

1. The time the patient spent with a photographer/imaging technician to complete the AS-OCT images, the time the patient spent with the optometrist to complete the LACD test and time the patient spent with the ophthalmologist to complete the gonioscopic examination. This information will be obtained in a consecutive group of patients until saturation is reached.
2. The time required by the optometrist, the ophthalmic photographer/imaging technician and the ophthalmologist to interpret the images and to determine whether there is angle closure or not.
3. Scores obtained in the health-related quality of life (HRQOL) questionnaire (EQ-5D-5L) filled in by patients and collected at the study visit, which will provide utility data for different health states.
4. Resource use data will be collected to explore the costs of delivering the standard care pathway and the new proposed triage pathway and to find the key cost drivers.

#### Trial management arrangements

The CI will have overall responsibility for the conduct of the study. The NICTU will undertake trial management including all clinical trial applications (Ethics and Research Governance), site initiation and training, monitoring and reports to ethics, Sponsor and Funder. The trial coordinator will be responsible on a day-to-day basis for overseeing and coordinating the work of the multidisciplinary trial team, and will be the main contact between the trial team and other parties involved. Before the trial starts, site training will take place to ensure that all relevant essential documents and trial supplies are in place and that site staff are fully aware of the trial protocol and procedures. The NICTU will assist and facilitate in the setting up and coordination of the trial committees.

#### Trial Management Group

A Trial Management Group (TMG) will be established and Chaired by the CI. The TMG will include representation from the NICTU and other investigators or collaborators who are involved in the study and provide trial specific expertise (eg, trial statistician, health economist). This group will have responsibility for the day-to-day operational management of the trial. Regular meetings of the TMG will be held to discuss and monitor progress. The discussions of the TMG will be formally minuted and a record kept in the Trial Master File. A TMG charter will be drawn up to detail the terms of reference of the TMG, including roles and responsibilities of the members.

#### Trial Steering Committee

The conduct of the trial will be overseen by an independent Trial Steering Committee (TSC). Throughout the study, the TSC will take responsibility for monitoring and guiding overall progress, scientific standards, operational delivery and protecting the rights and safety of patients enrolled in the study. The TSC will include an independent statistician, a health economist, at least two independent clinicians and a patient representative. The CI will attend the TSC meetings. Representatives of the Sponsor, Funder and the NICTU may attend TSC meetings as observers and at the discretion of the Chair. The TSC Charter will outline the terms of reference of the TSC including roles and responsibilities, membership, organisation of meetings (including frequency), reporting, decision-making and the relationship with the other trial committees.

#### Data Monitoring and Ethics Committee

The main role of the Data Monitoring and Ethics Committee (DMEC) will be to monitor the trial data and make recommendations to the TSC on whether there are any ethical or safety reasons why the trial should not continue. The DMEC will include a researcher, a statistician and a clinician. Attendance at DMEC meetings by non-members will be at the discretion of the Chair. The primary DMEC reporting line will be via the Chair to the TSC. The DMEC Charter will outline the terms of reference of the DMEC including roles and responsibilities, membership, organisation of meetings (including frequency), reporting, decision-making and the relationship with the other trial committees.

### Patient and public involvement

The patient and public involvement (PPI) co-applicants will be supported to convene a PPI group that will actively contribute and advise on all patient-facing and public-facing documentation, including promotional patient information leaflets, informed consent forms (ICF) and plain language summaries. They will also be involved in dissemination of the study’s results to patients and the public. The PPI group will meet during the development phase, at the end of the internal pilot phase (when the progression criteria are being considered) and at the end of the study.

#### Protocol compliance

A protocol deviation is defined as an incident which deviates from the normal expectation of a particular part of the study process. Any deviations from the protocol will be fully documented on the protocol deviation form in the CRF.

A serious breach is defined as a deviation from the study protocol or GCP which is likely to effect to a significant degree:

1. The safety or physical or mental integrity of the patients in the trial; or
2. The scientific value of the trial.

The PI or designee is responsible for ensuring that serious breaches are reported directly to the NICTU within one working day of becoming aware of the breach, using the dedicated email address (clinicaltrials@nictu.hscni.net). The NICTU will notify the CI and Sponsor immediately to ensure adherence to the reporting requirements to the Research Ethics Committee (REC) where a serious breach has occurred. Protocol compliance will be monitored by the NICTU who will undertake site visits to ensure that the trial protocol is adhered to and that necessary paperwork (eg, CRFs, patient consent) is being completed appropriately.

The trial has been registered with ISCTRN (ISRCTN registry – 43107).

### Statistical considerations

#### Sample size

We will recruit 600 individuals who have been referred with suspected angle closure from primary care (community optometry). According to our feasibility work, we estimate that approximately half of these 600 individuals will have angle closure and that half will not have it. Angle closure is typically a bilateral disease, and, thus, the majority of individuals will have similar angle characteristics in both eyes but, in the rare case of an individual having angle closure in only one eye, they will be considered to have angle closure.

Previous studies have shown that using a cut-off to capture 90% sensitivity corresponds to a specificity of around 75% with AS-OCT or with LACD.^14–16^ Therefore the study will have a 95% probability of detecting the true sensitivity of either test to within ±3.5% (ie, the CI for the true sensitivity would be approximately 7% points in width), based on a sensitivity of 90%. The study would also have a 95% probability of detecting the true specificity of either test to within ±5% (ie, the CI for the true specificity would be 10% in width), assuming a true specificity of 75%. These sample sizes are conservative because they are based on using only one eye per person, while in practice, as described below, information from both eyes will be used.

#### Statistical analysis

Sensitivity and specificity will be calculated using data from both eyes, CIs will then be calculated using variance inflation factors to account for the lack of independence of each eye in the same person (using the svy function in Stata).^17^ Primary analysis will be based on a binary result, closed or not closed. A sensitivity analysis will be conducted calculating test performance measures using a multilevel logistic regression model, with eyes nested within person and person as a random effect. We will extend this model to include the site and operator of the various tests to explore the impact these have on the width of the CIs for sensitivity and specificity.

Secondary analyses will be conducted investigating different degrees of angle closure (on LACD and AS-OCT). Initially, a cut-off will be selected to obtain a sensitivity of 90% and the resulting specificity will be determined. We will explore the diagnostic accuracy of combining both tests (LACD and AS-OCT), and of using different thresholds for a positive result. We will also compare the diagnostic accuracy of AS-OCT images interpreted by optometrists, photographers/imaging technicians and ophthalmologists.

#### Health economics analysis

We will evaluate LACD and AS-OCT as part of a triage test to diagnose angle closure in patients referred to HES with possible glaucoma.

We propose to use a Markov model to assess the longer-term costs and effects of alternate diagnostic pathways. We propose to run this over an expected lifetime horizon. We propose to adapt a model developed and published by members of the study team.^18^ The model comprises six states—normal vision, suspected glaucoma, glaucoma without blindness, glaucoma-related unilateral blindness, glaucoma-related bilateral blindness and death. Accurate testing may delay progression, through earlier identification and treatment. Transition probabilities, costs and outcomes (beyond those observed in the trial) associated with the various states will be taken from the literature, supplemented where appropriate with expert opinion and uncertainty explored using sensitivity analyses.

#### Three tests will be used in the study

Gonioscopy as reference standard; LACD; AS-OCT and concordant LACD/AS-OCT results as triage tests. Three comparison groups will be created based on data collected: (1) comparison of gonioscopy versus LACD only; (2) comparison of gonioscopy versus AS-OCT only; and (3) comparison of gonioscopy versus LACD and AS-OCT concordant responses. The proportion of accurate diagnoses across modalities using gonioscopy as reference standard will provide a measure of effect and the differences in these will provide a measure of the incremental effect.

An NHS healthcare perspective will be used to evaluate cost-effectiveness. For the cost-utility analysis, EQ-5D-5L data will also be gathered at baseline and provide the ‘starting point’ against which subsequent decrements in HRQOL arising from deteriorating vision will be compared. The trajectory of HRQOL after ‘normal vision’ will be based on estimates from a systematic review of the literature for the health states specified in the Markov model. As HRQOL will vary based on age and time spent in a given state, adjustments for these will be made to reflect changes associated with cohort ageing over repeated cycles of the model up to termination (death) for all members.

A similar approach will be adopted with respect to costs. In both cases a survey of experts will be used to address gaps in estimates and one-way and probabilistic sensitivity analysis will be used to explore uncertainty in such estimates. It is anticipated that cycle length will be 1 year.

The alternative test arrangements will have differential costs associated with them—for example, gonioscopy for all referrals likely being more expensive than those triaged based on LACD only or LACD only being less expensive than those triaged on a combination of LACD and AS-OCT. They may also have differential effects in terms of identification for further investigation and treatment. While we anticipate false negatives/positives will be identified quickly without adverse health effects, the model will allow us to examine differential costs over a hypothetical lifetime for the cohort as well as to explore scenarios in which delayed identification does result in adverse effects.

Incremental quality-adjusted life year (QALYs) and costs will be estimated for cohorts across triage and gonioscopy tests and expressed in terms of cost per QALY gain. Uncertainty around the threshold willingness to pay for a QALY will be explored using cost-effectiveness acceptability curves. Costs and outcomes will be discounted at 3.5% per annum as per current National Institute for health and Clinical Excellence (NICE) guidance (https://www.nice.org.uk/process/pmg6/chapter/assessing-cost-effectiveness). Subgroup analyses will examine potential differences in ICERs (Incremental Cost-Effectiveness Ratios) across groups differentiated by age at screening sex and ethnic groups. Reporting of results will adhere to revised-Consolidated Health Economic Evaluation Reporting Standards checklist and the International Society for Pharmacoeconomics and Outcomes Research (ISPOR) modelling good practice.^19^

## Ethics and dissemination

The study will be conducted in accordance with the ethical principles that have their origin in the Declaration of Helsinki. Eligible patients will only be included in the trial after written informed consent is obtained. Informed consent must be obtained prior to conducting any trial specific procedures and the process for obtaining informed consent must be documented in the patient’s medical records (source documents will be reviewed at the time of on-site monitoring visits).

ICF approved by the REC will be provided by the NICTU. The PI or designee is responsible for ensuring that informed consent for trial participation is given by each patient prior to any trial procedure being performed. This requires that the ICF be signed and personally dated by the patient prior to any trial procedures being undertaken. If no consent is given, a patient cannot be recruited into the trial. Two copies of the ICF must be signed and personally dated by the patient and the individual taking consent. A copy of the signed ICF will be filed in the patient’s medical records, while the originals will be retained by the patient and by the PI in the Investigator Site File.

Queen’s University Belfast (QUB) will act as Sponsor for the study and the CI will take overall responsibility for the conduct of the study (https://www.qub.ac.uk/Research/Research-contacts/). Separate agreements will be put in place between the Sponsor and each organisation who will undertake Sponsor delegated duties in relation to the management of the study.

The protocol has been approved by an Ethical Review Board, the London—City and East Research Ethics Committee: REC reference:22/LO/0885, IRAS project ID: 315388. Changes to the protocol may require ethics committee approval or favourable opinion prior to implementation. The NICTU in collaboration with the sponsor will submit all protocol modifications to the REC for review in accordance with the governing regulations.

In order to maintain confidentiality, all CRFs, questionnaires, study reports and communication regarding the study will identify the patients by their assigned unique trial identifier only. Computers where information will be stored will be password protected. Patient confidentiality will be maintained at every stage and will not be made publicly available to the extent permitted by the applicable laws and regulations.

Regarding dissemination, it is anticipated that the study findings will be published in national and international peer-review journals and these articles will be led by the CI. This will secure a searchable compendium of these publications and make the results readily accessible to the public and healthcare professionals. In addition, study findings may be presented at both national and international meetings and to appropriate patient groups.

A report containing the methodology and results of this diagnostic study will be published as a Health Technology Assessment (HTA) monograph, freely accessible via the National Institute of Health Research HTA web page. The Royal College of Ophthalmologist will be contacted once the study is completed to allow the trial’s findings to be incorporated in future glaucoma guidelines.

### Data access

Following the publication of the primary and secondary outcomes, there may be scope to conduct additional analyses on the data collected. In such instances, formal requests for data will need to be made in writing to the CI who will discuss this with the TMG and obtain approval from the Sponsor. In the event of publications arising from such analyses, those responsible will need to provide the CI with a copy of any intended manuscript for approval prior to submission. Authorship will need to take the format of ‘[name] on behalf of the ACE Clinical Trial Group’ or something similar, which will be agreed by the TMG.

## Supplementary Material

Reviewer comments

Author's manuscript
