# Supplementary material for: Technologies for the diagnosis of angle closure glaucoma (ACE): protocol of a prospective, multicentre, cross-sectional diagnostic study
Source: BMJ Open. 2023 Oct 4;13(10):e073975. doi: 10.1136/bmjopen-2023-073975 (PMC10551982; doi:10.1136/bmjopen-2023-073975)
Supplement: Supplementary data [file bmjopen-2023-073975supp001.pdf]

Appendix 1. Schedule of Assessments

Patients will be required to attend one study visit (Baseline/Visit 1). At this visit, eligibility will be confirmed, consent obtained and all study procedures will be completed.

Baseline/Visit 1

Step 1

Confirmation of eligibility by an ophthalmologist.

Written patient informed consent obtained to participate in the ACE study.

Step 2

Patients will undergo the following tests:

- AS-OCT, by an ophthalmic photographer/imaging technician
- Limbal anterior chamber depth (LACD), by a hospital optometrist
- Gonioscopy, by an ophthalmologist glaucoma expert

Step 3

Patients will complete the EQ-5D-5L questionnaire.

Step 4

The patients' information obtained during the standard care pathway will be recorded in the CRF including details on:

- Demographics and postal code
- Relevant medical and ophthalmic history
- Best corrected visual Acuity
- Refractive error

- Intraocular pressure
- Details on the presence/absence of glaucomatous damage
- If there is glaucomatous damage the MD value of the visual field test and reliability (percentage of false positives) will be recorded, if available.
- Any significant ocular co-morbidity

All ocular data will be obtained for both eyes separately.

Step 5

AS-OCT scans will be anonymised and transferred to QUB reading centre where they will be uploaded to an electronic website developed for the ACE study. The reading centre will then create folders and make these anonymised images accessible to the optometrists, ophthalmic photographers/imaging technicians and ophthalmologists.

Step 6

AS-OCT images will be interpreted by optometrists, ophthalmic photographers/technicians and ophthalmologists, masked to the reference standard (gonioscopy by ophthalmologists).

Optometrists, ophthalmic photographers/imaging technicians and ophthalmologists reading the images will not evaluate images of patients from their own institution to assure masking (see below). Once the images are read, the optometrists, ophthalmic photographers/imaging technicians and ophthalmologists will determine:

- whether there is angle closure or not
- whether they are unsure and reasons (e.g., poor image quality)

The optometrists, ophthalmic photographers/imaging technicians and ophthalmologists will record this information in the appropriate CRF.

BMJ Publishing Group Limited (BMJ) disclaims all liability and responsibility arising from any reliance placed on this supplemental material which has been supplied by the author(s)
