# Supplementary material for: Technologies for the diagnosis of angle closure glaucoma (ACE): protocol of a prospective, multicentre, cross-sectional diagnostic study
Source: BMJ Open. 2023 Oct 4;13(10):e073975. doi: 10.1136/bmjopen-2023-073975 (PMC10551982; doi:10.1136/bmjopen-2023-073975)
Supplement: Author's manuscript [file bmjopen-2023-073975.draft_revisions.pdf]

BMJ Open is committed to open peer review. As part of this commitment we make the peer review history of every article we publish publicly available.

When an article is published we post the peer reviewers' comments and the authors' responses online. We also post the versions of the paper that were used during peer review. These are the versions that the peer review comments apply to.

The versions of the paper that follow are the versions that were submitted during the peer review process. They are not the versions of record or the final published versions. They should not be cited or distributed as the published version of this manuscript.

BMJ Open is an open access journal and the full, final, typeset and author-corrected version of record of the manuscript is available on our site with no access controls, subscription charges or pay-per-view fees (http://bmjopen.bmj.com).

If you have any questions on BMJ Open's open peer review process please email info.bmjopen@bmj.com

Technologies for the Diagnosis of Angle Closure glaucoma (ACE). Protocol of a prospective, multi-centre, cross-sectional diagnostic study.

Journal:	*BMJ Open*
Manuscript ID	bmjopen-2023-073975
Article Type:	Protocol
Date Submitted by the Author:	24-Mar-2023

Complete List of Authors:	Azuara-Blanco, Augusto ; Queen's University Belfast, Centre for Public Health<br>Lawrenson, John j.g.; City University of London<br>King, Anthony ; Nottingham University Hospitals NHS Trust, Department of Ophthalmology<br>Foster, Paul; University College London, Institute of Ophthalmology. Division of of Epidemiology<br>Virgili, Gianni ; Queen's University Belfast, Centre for Public Health<br>Guiney, Mary; Belfast Health and Social Care Trust, Northern Ireland Clinical Trials Unit<br>Nolan, Winnie; Moorfields Eye Hospital City Road Campus, Glaucoma Service<br>Jindal, Anish; Moorfields Eye Hospital City Road Campus<br>Sharma, Meenakshi; Queen's University Belfast, Centre for Public Health<br>O'Neill, Ciaran; Queen's University Belfast, Centre for Public Health<br>Cardwell, Christopher; Queen's University Belfast, School of Medicine, Dentistry and Biomedical Sciences<br>Clarke, Mike; Queen's University Belfast, Northern Ireland Methodology Hub

Keywords:	OPHTHALMOLOGY, Glaucoma < OPHTHALMOLOGY, Clinical Trial, Diagnostic Imaging

SCHOLARONE™
Manuscripts

Technologies for the Diagnosis of Angle Closure glaucoma (ACE). Protocol of a prospective, multi-centre, cross-sectional diagnostic study.

- Augusto Azuara-Blanco; Centre for Public Health, Queen's University Belfast
- John JG Lawrenson; Department of Optometry and Visual Sciences, City University of London
- Anthony King; Department of Ophthalmology, Nottingham University Hospitals NHS Trust
- Paul Foster; Institute of Ophthalmology. Division of Epidemiology, University College London
- Gianni Virgili; Centre for Public Health, Queen's University Belfast
- Mary Guiney; Northern Ireland Clinical Trials Unit, Belfast Health and Social Care Trust
- Winnie Nolan; Glaucoma Service, Moorfields Eye Hospital City Road Campus
- Anish Jindal; Glaucoma Service, Moorfields Eye Hospital City Road Campus
- Meenakshi Sharma; Centre for Public Health, Queen's University Belfast
- Ciaran O'Neill; Centre for Public Health, Queen's University Belfast
- Christopher Cardwell; Centre for Public Health, Queen's University Belfast
- Mike Clarke; Centre for Public Health, Queen's University Belfast

Corresponding author: Augusto Azuara-Blanco (a.azuara-blanco@qub.ac.uk)

Conflicts of interest: none

For peer review only - http://bmjopen.bmj.com/site/about/guidelines.xhtml

Abstract

Introduction: Angle-closure is responsible for half of all glaucoma blindness globally. Patients with suspected glaucoma require assessment of the drainage angle by an experienced clinician. The goal of this study is to evaluate the diagnostic performance and cost-effectiveness of two non-contact tests, anterior segment OCT (AS-OCT) and limbal anterior chamber depth (LACD) for patients referred to hospital with suspected angle closure compared to gonioscopy by ophthalmologist.

Methods and Analysis. Study design: prospective, multi-centre, cross-sectional diagnostic accuracy study. Inclusion criteria: adults referred from community optometry to hospital with suspected angle closure.

Primary outcome: Sensitivity and specificity. Secondary outcomes: Positive/negative likelihood ratios, concordance, cost -effectiveness, proportion of patients requiring subsequent clinical assessment by ophthalmologist.

Sample size: 600 individuals who have been referred with suspected angle closure from primary care (community optometry). We will have a 95% probability of detecting the true sensitivity of either test to within ±3.5% based upon a sensitivity of 90%. The study would also have a 95% probability of detecting the true specificity of either test to within ±5%, assuming a specificity of 75%.

Ethics and Dissemination: Ethical Review Board approval was obtained. REC reference: 22/LO/0885. Our findings will be disseminated to those involved in eye care services. We will have a knowledge exchange event at the end of the study, publish via the Health Technology Assessment webpage and in specialist journals. The results will be presented at professional conferences and directly to patients via patient group meetings and the Glaucoma UK charity.

Registration: ISRCTN registry – 43107

For peer review only

Keywords: angle-closure glaucoma; diagnosis; anterior-segment optical coherence tomography; limbal anterior chamber depth; gonioscopy

Word count: 3791

Article summary:

- Protocol of a diagnostic study on angle closure (ACE)
- Study Design: prospective, cross-sectional, multi-centre
- Masked investigators will evaluate the following non-contact tests, anterior segment OCT and limbal anterior chamber depth, compared with a reference standard, gonioscopy by expert ophthalmologist
- Study setting: hospital eye services.
- Strengths and limitations of this study. Strengths: prospective study design; masked investigators; adequate, large sample size; evaluation of cost-effectiveness. Limitations: subjective reference standard

For peer review only

Introduction

Glaucoma is a chronic optic neuropathy and a leading cause of irreversible blindness.[1-4] In the UK, more than 500,000 people have glaucoma, and 4000 new patients are registered each year with sight loss because of glaucoma . Many more people have glaucoma not severe enough to be registered, but severe enough to reduce vision and quality of life (e.g., loss of their driving licence).[5]

Hospital eye services (HES) account for 8% of outpatient attendances to the NHS and glaucoma accounts for 25% of outpatient activity of hospital eye services, with more than 1 million visits per year in England alone due to glaucoma.[5]

There are two types of glaucoma, according to the appearance of the drainage angle of the eye: open angle glaucoma (OAG), the most common, and angle closure glaucoma (ACG). In ACG, which is the subject of this study, the drainage angle is blocked, leading to acute or chronic elevation of intraocular pressure (IOP) and damage of the optic nerve.

ACG is less common but more severe than OAG and its prevalence increases with age [Bourne, Tham]. Acute angle closure is an uncommon but serious ocular emergency.[6,7] In the early stage of the disease, angle closure can be treated with laser to reduce the risk of developing glaucoma and vision loss.[6,8]

ACG is responsible for approximately 1 in 6 glaucomas in the UK and a substantial proportion of glaucoma referrals to hospital eye services.[9,10]

A recent Cochrane review found sub-optimal quality evidence regarding the diagnostic performance of non-contact tests, AS-OCT and LACD.[11] Pooled data showed that LACD had high sensitivity and a possibly sufficient specificity for case finding and performed as well as AS-OCT but the authors highlighted "There is still a need for high-quality studies to evaluate the performance of non-contact tests for angle assessment." Given the demands OAG presents the health service and the implications delays in diagnosis may have for patients, clearly it is important to assess the cost-effectiveness of potential improvements to

For peer review only - http://bmjopen.bmj.com/site/about/guidelines.xhtml

the diagnostic pathway. Our study will fill this gap and determine the potential of these tests in the diagnosis of angle closure.

Our hypothesis is that the non-contact tests for diagnosing angle closure will be accurate and will facilitate a safe and efficient pathway for patients with this condition. The goal of this study is to evaluate the diagnostic performance and cost-effectiveness of two non-contact tests, anterior segment OCT (AS-OCT) and limbal anterior chamber depth (LACD) for patients referred to hospital with suspected angle closure compared to standard practice (gonioscopy by ophthalmologist). This report describes the protocol version 1.0.

Methods and analysis:

Study Design: ACE is a prospective, cross-sectional, multi-centre, diagnostic accuracy study of people referred to hospital eye services with suspected angle closure. Study setting is secondary care, hospital eye services.

Sampling and data collection will be carried out prospectively. Consecutive eligible patients will be approached to take part in the study (Figure 1). Patients referred to hospital eye services with suspected angle closure will be approached and those who consent will undergo testing with the two non-contact technologies (index tests) on the same day. Patients will undergo AS-OCT (with any device available at the site) and the images of the temporal and nasal angle will be sent to a reading centre for interpretation. Interpretation of AS-OCT will use reference images and the investigator will determine if the angle is closed (i.e., contact between peripheral iris and trabecular meshwork at one or both quadrants), open or indeterminate. In addition the investigator will grade the angle opening (<10 degrees, 10-20 degrees or >degrees) using reference images. The LACD will be performed and graded by a hospital optometrist (masked to the AS-OCT and to the reference standard). The investigator will use reference images to determine if the angle is closed (i.e., contact between peripheral iris and cornea), open, or indeterminate. The investigator will also grade the angle opening according to a 7-point scale proposed by Foster et al, using reference images.[12] Patients will then receive the reference standard (gonioscopy),

For peer review only - http://bmjopen.bmj.com/site/about/guidelines.xhtml

provided by an expert who will be masked to the evaluated tests (LACD and AS-OCT). The expert will be an ophthalmologist with glaucoma expertise and will judge if the angle is open or closed. Masked ophthalmologists, optometrists and trained photographers/imaging technicians will interpret AS-OCTs images provided by the reading centre at a different time. Prior the study recruitment we will meet clinicians and investigators at each study site to review and agree on the reference standard and interpretation of tests.

Inclusion criteria: Adults (≥18 years) referred from community optometry to hospital eye services with suspected angle closure, with or without additional co-morbidity. Exclusion criteria: Unable to provide informed consent; unable to undergo a slit-lamp examination.

The primary outcome is sensitivity and specificity of the non-contact tests to detect angle-closure. Reference standard is gonioscopy by an ophthalmologist with glaucoma expertise. Secondary outcomes: Positive/negative likelihood ratios, concordance, cost-effectiveness, proportion of patients requiring subsequent clinical assessment by ophthalmologist, proportion of patients unable to undergo tests and of tests of inadequate quality.

Recruitment of study participants: Potential patients will be identified through referral letters from optometrists to hospital eye services. Potential patients with possible angle closure will be approached before they come for their routine clinical appointment, by phone or via an invitation letter.

Prior to the initiation of the study, training will be provided to the investigators on the interpretation and grading of the tests (AS-OCT, LACD, gonioscopy) via a web-based teaching module on AS-OCT images and LACD interpretation.

The optometrists performing the LACD test will masked to gonioscopy. Optometrists, ophthalmic photographers/imaging technicians and ophthalmologists interpreting patients' images will be masked to the reference standard.

For peer review only

Ophthalmologists performing the gonioscopic evaluation will also be masked to the optometrist LACD test result and to the AS-OCT test, including the findings/decisions made by the optometrists, ophthalmic photographers/imaging technicians and ophthalmologists (who will be reviewing the images at a later date). At the same visit participants will be asked to complete a health status questionnaire, EQ-5D-5L.[13]

Data collection and management: the Chief Investigator (CI) and NICTU will provide training to site staff on trial processes and procedures, including the completion of the case report form (CRF) and data collection. Monitoring during the trial will check the accuracy of entries on CRF's against the source documents, the adherence to the protocol, procedures and GCP, as outlined in the trial monitoring plan.

Quality control is implemented by the NICTU in the form of Standard Operating Procedures (SOPs), which are defined to encompass aspects of the clinical data management process, and to ensure standardisation and adherence to International Conference of Harmonisation Good Clinical Practice (ICH-GCP) guidelines and regulatory requirements.

Data validation will be implemented and discrepancy reports will be generated following data entry to identify discrepancies such as out of range, inconsistencies or protocol deviations based on data validation checks programmed in the clinical trial database.

To ensure accurate, complete and reliable data are collected, the NICTU will provide training to site staff either through investigator meetings or site initiation visits.

All data for an individual patient will be collected by the PI or designee and recorded in source documents and/or the CRF for the study. The EQ-5D-5L questionnaires will be completed by the patient. Patient identification on the CRF and questionnaires will be through their unique trial identifier, allocated at the time of recruitment.

Case report forms and questionnaires are to be submitted to the NICTU as per the CRF submission schedule.

In addition to the data specified in the sections above, the following information will be obtained and recorded in the appropriate CRF:

For peer review only

1) The time the patient spent with a photographer/imaging technician to complete the AS-OCT images, the time the patient spent with the optometrist to complete the LACD test, and time the patient spent with the ophthalmologist to complete the gonioscopic examination. This information will be obtained in a consecutive group of patients until saturation is reached.
2) The time required by the optometrist, the ophthalmic photographer/imaging technician and the ophthalmologist to interpret the images and to determine whether there is angle closure or not.
3) Scores obtained in the health related quality of life questionnaire (EQ-5D-5L) filled in by patients and collected at the study visit, which will provide utility data for different health states.
4) Resource use data will be collected to explore the costs of delivering the standard care pathway and the new proposed triage pathway and to find the key cost drivers.

Trial Management Arrangements: the CI will have overall responsibility for the conduct of the study. The NICTU will undertake trial management including all clinical trial applications (Ethics and Research Governance), site initiation and training, monitoring, and reports to ethics, Sponsor and Funder. The trial co-ordinator will be responsible on a day to day basis for overseeing and co-ordinating the work of the multi-disciplinary trial team, and will be the main contact between the trial team and other parties involved. Before the trial starts, site training will take place to ensure that all relevant essential documents and trial supplies are in place and that site staff are fully aware of the trial protocol and procedures. The NICTU will assist and facilitate in the setting up and co-ordination of the trial committees.

Trial Management Group (TMG): a TMG will be established and Chaired by the CI. The TMG will include representation from the NICTU and other investigators or collaborators who are involved in the study and provide trial specific expertise (e.g. trial statistician, health economist). This group will have responsibility for the day to day operational management of the trial. Regular meetings of the TMG will be held to discuss and monitor progress. The

For peer review only - http://bmjopen.bmj.com/site/about/guidelines.xhtml

discussions of the TMG will be formally minuted and a record kept in the Trial Master File. A TMG Charter will be drawn up to detail the terms of reference of the TMG, including roles and responsibilities of the members.

Trial Steering Committee (TSC): the conduct of the trial will be overseen by an independent TSC. Throughout the study, the TSC will take responsibility for monitoring and guiding overall progress, scientific standards, operational delivery and protecting the rights and safety of patients enrolled in the study. The TSC will include an independent statistician, a health economist, at least two independent clinicians and a patient representative. The CI will attend the TSC meetings. Representatives of the Sponsor, Funder and the NICTU may attend TSC meetings as observers and at the discretion of the Chair. The TSC Charter will outline the terms of reference of the TSC including roles and responsibilities, membership, organisation of meetings (including frequency), reporting, decision making and the relationship with the other trial committees.

Data Monitoring and Ethics Committee (DMEC): the main role of the DMEC will be to monitor the trial data and make recommendations to the TSC on whether there are any ethical or safety reasons why the trial should not continue. The DMEC will include a researcher, a statistician and a clinician. Attendance at DMEC meetings by non-members will be at the discretion of the Chair. The primary DMEC reporting line will be via the Chair to the TSC. The DMEC Charter will outline the terms of reference of the DMEC including roles and responsibilities, membership, organisation of meetings (including frequency), reporting, decision making and the relationship with the other trial committees.

Patient and Public Involvement (PPI): the PPI co-applicants will be supported to convene a PPI Group that will actively contribute and advise on all patient- and public-facing documentation, including promotional patient information leaflets, informed consent forms and plain language summaries. They will also be involved in dissemination of the study's results to patients and the public. The PPI group will meet during the development phase, at

For peer review only - http://bmjopen.bmj.com/site/about/guidelines.xhtml

the end of the internal pilot phase (when the progression criteria are being considered) and at the end of the study.

Protocol Compliance: a protocol deviation is defined as an incident which deviates from the normal expectation of a particular part of the study process. Any deviations from the protocol will be fully documented on the protocol deviation form in the CRF.

A serious breach is defined as a deviation from the study protocol or GCP which is likely to effect to a significant degree:

(a) the safety or physical or mental integrity of the patients in the trial; or

(b) the scientific value of the trial

The PI or designee is responsible for ensuring that serious breaches are reported directly to the NICTU within one working day of becoming aware of the breach, using the dedicated email address (clinicaltrials@nictu.hscni.net). The NICTU will notify the CI and Sponsor immediately to ensure adherence to the reporting requirements to REC where a serious breach has occurred. Protocol compliance will be monitored by the NICTU who will undertake site visits to ensure that the trial protocol is adhered to and that necessary paperwork (e.g. CRFs, patient consent) is being completed appropriately.

The trial has been registered with ISCTRN (ISRCTN registry – 43107).

Statistical considerations

Sample size: we will recruit 600 individuals who have been referred with suspected angle closure from primary care (community optometry). According to our feasibility work, we estimate that approximately half of these 600 individuals will have angle closure and that half will not have it. Angle closure is typically a bilateral disease, and, thus, the majority of individuals will have similar angle characteristics in both eyes but, in the rare case of an individual having angle closure in only one eye, they will be considered to have angle closure.

For peer review only

Previous studies have shown that using a cut off to capture 90% sensitivity corresponds to a specificity of around 75% with AS-OCT or with LACD.[14-16] Therefore the study will have a 95% probability of detecting the true sensitivity of either test to within ±3.5% (i.e. the confidence interval for the true sensitivity would be approximately 7 percentage points in width), based upon a sensitivity of 90%. The study would also have a 95% probability of detecting the true specificity of either test to within ±5% (i.e., the confidence interval for the true specificity would be 10% in width), assuming a true specificity of 75%. These sample size are conservative because they are based on using only one eye per person, while in practice, as described below, information from both eyes will be used.

Statistical analysis: sensitivity and specificity will be calculated using data from both eyes, confidence intervals will then be calculated using variance inflation factors to account for the lack of independence of each eye in the same person (using the svy function in STATA).[17] Primary analysis will be based upon a binary result, closed or not closed. A sensitivity analysis will be conducted calculating test performance measures using a multi-level logistic regression model, with eyes nested within person, and person as a random effect. We will extend this model to include site and operator of the various tests to explore the impact these have on the width of the confidence intervals for sensitivity and specificity.

Secondary analyses will be conducted investigating different degrees of angle closure (on LACD and AS-OCT). Initially, a cut-off will be selected to obtain a sensitivity of 90% and the resulting specificity will be determined. We will explore the diagnostic accuracy of combining both tests (LACD and AS-OCT), and of using different thresholds for a positive result. We will also compare the diagnostic accuracy of AS-OCT images interpreted by optometrists, photographers/imaging technicians and ophthalmologists.

Health economics analysis: we will evaluate LACD and AS-OCT as part of a triage test to diagnose angle closure in patients referred to hospital eye services with possible glaucoma. We propose to use a Markov model to assess the longer term costs and effects of alternate diagnostic pathways. We propose to run this over an expected lifetime horizon. We propose to adapt a model developed and published by members of the study team.[18] The model

For peer review only - http://bmjopen.bmj.com/site/about/guidelines.xhtml

comprises 6 states – normal vision, suspected glaucoma, glaucoma without blindness, glaucoma-related unilateral blindness, glaucoma-related bilateral blindness and dead. Accurate testing may delay progression, through earlier identification and treatment. Transition probabilities, costs and outcomes (beyond those observed in the trial) associated with the various states will be taken from the literature, supplemented where appropriate with expert opinion and uncertainty explored using sensitivity analyses.

Three tests will be used in the study: gonioscopy as reference standard; LACD; AS-OCT and concordant LACD/AS-OCT results as triage tests. Three comparison groups will be created based on data collected: (A) comparison of gonioscopy versus LACD only; (B) comparison of gonioscopy versus AS-OCT only; and (C) comparison of gonioscopy versus LACD and AS-OCT concordant responses. The proportion of accurate diagnoses across modalities using gonioscopy as reference standard will provide a measure of effect and the differences in these will provide a measure of the incremental effect.

An NHS healthcare perspective will be used to evaluate cost effectiveness. For the cost-utility analysis, EQ-5D-5L data will also be gathered at baseline and provide the "starting point" against which subsequent decrements in health-related quality of life (HRQOL) arising from deteriorating vision will be compared. The trajectory of HRQOL after "normal vision" will be based on estimates from a systematic review of the literature for the health states specified in the Markov model. As HRQOL will vary based on age and time spent in a given state, adjustments for these will be made to reflect changes associated with cohort aging over repeated cycles of the model up to termination (death) for all members.

A similar approach will be adopted with respect to costs. In both cases a survey of experts will be used to address gaps in estimates and one-way and probabilistic sensitivity analysis will be used to explore uncertainty in such estimates. It is anticipated that cycle length will be one year.

The alternative test arrangements will have differential costs associated with them – for example, gonioscopy for all referrals likely being more expensive than those triaged based on LACD only or LACD only being less expensive than those triaged on a combination of

For peer review only - http://bmjopen.bmj.com/site/about/guidelines.xhtml

LACD and AS-OCT. They may also have differential effects in terms of identification for further investigation and treatment. While we anticipate false negatives/positives will be identified quickly without adverse health effects, the model will allow us to examine differential costs over a hypothetical lifetime for the cohort as well as to explore scenarios in which delayed identification does result in adverse effects.

Incremental QALYs and costs will be estimated for cohorts across triage and gonioscopy tests and expressed in terms of cost per QALY gain. Uncertainty around the threshold willingness to pay for a QALY will be explored using cost-effectiveness acceptability curves. Costs and outcomes will be discounted at 3.5% per annum as per current NICE guidance (https://www.nice.org.uk/process/pmg6/chapter/assessing-cost-effectiveness). Sub-group analyses will examine potential difference in ICERs across groups differentiated by age at screening sex and ethnic group. Reporting of results will adhere to revised-CHEERS checklist and ISPOR modelling good practice.[19]

Ethics and dissemination

The study will be conducted in accordance with the ethical principles that have their origin in the Declaration of Helsinki. Eligible patients will only be included in the trial after written informed consent is obtained. Informed consent must be obtained prior to conducting any trial specific procedures and the process for obtaining informed consent must be documented in the patient's medical records (source documents will be reviewed at the time of on-site monitoring visits).

Informed Consent Forms (ICF) approved by the REC will be provided by the NICTU. The PI or designee is responsible for ensuring that informed consent for trial participation is given by each patient prior to any trial procedure being performed. This requires that the ICF be signed and personally dated by the patient prior to any trial procedures are undertaken. If no consent is given, a patient cannot be recruited into the trial. Two copies of the ICF must be signed and personally dated by the patient and the individual taking consent. A copy of the

For peer review only - http://bmjopen.bmj.com/site/about/guidelines.xhtml

signed ICF will be filed in the patient's medical records, whilst the originals will be retained by the patient and by the PI in the Investigator Site File (ISF).

Queen's University Belfast (QUB) will act as Sponsor for the study and the CI will take overall responsibility for the conduct of the study (https://www.qub.ac.uk/Research/Research-contacts/). Separate agreements will be put in place between the Sponsor and each organisation who will undertake Sponsor delegated duties in relation to the management of the study.

Funding: funding was obtained from the National Institute of Health Research (NIHR) Health Technology Assessment Programme (HTA). Project ID: HTA NIHR134593. The views expressed are those of the author(s) and not necessarily those of the NIHR or the Department of Health and Social Care.

The protocol has been approved by an Ethical Review Board: REC reference:22/LO/0885, IRAS project ID: 315388. Changes to the protocol may require ethics committee approval or favourable opinion prior to implementation. The NICTU in collaboration with the sponsor will submit all protocol modifications to the REC for review in accordance with the governing regulations.

In order to maintain confidentiality, all CRFs, questionnaires, study reports and communication regarding the study will identify the patients by their assigned unique trial identifier only. Computers where information will be stored will be password protected. Patient confidentiality will be maintained at every stage and will not be made publicly available to the extent permitted by the applicable laws and regulations.

Regarding dissemination, it is anticipated that the study findings will be published in national and international peer review journals and these articles will be led by the CI. This will secure a searchable compendium of these publications and make the results readily accessible to the public and health care professionals. In addition, study findings may be presented at both national and international meetings and to appropriate patient groups.

A report containing the methodology and results of this diagnostic study will be published as a Health Technology Assessment monograph, freely accessible via the NIHR HTA webpage.

For peer review only - http://bmjopen.bmj.com/site/about/guidelines.xhtml

The Royal College of Ophthalmologist will be contacted once the study is completed to allow the trial’s findings to be incorporated in future glaucoma guidelines.

Data Access

Following the publication of the primary and secondary outcomes, there may be scope to conduct additional analyses on the data collected. In such instances, formal requests for data will need to be made in writing to the CI who will discuss this with the TMG and obtain approval from the Sponsor. In the event of publications arising from such analyses, those responsible will need to provide the CI with a copy of any intended manuscript for approval prior to submission. Authorship will need to take the format of “[name] on behalf of the ACE Clinical Trial Group” or something similar, which will be agreed by the TMG.

Author contributions. All authors made a substantive intellectual contribution to the study design and the report.

Acknowledgements

Tara Holland, Ruth McCoy and Glaucoma UK for PPI support and advice .

For peer review only

References

1. Tham YC, Li X, Wong TY, et al. Global prevalence of glaucoma and projections of glaucoma burden through 2040: a systematic review and meta-analysis. Ophthalmology. 2014; 121:2081-90.

2. Jonas JB, Aung T, Bourne RR, et al. Glaucoma. Lancet. 2017;390(10108):2183-2193.

3. Quartilho A, Simkiss P, Zekite A, Xing W, Wormald R, Bunce C. Leading causes of certifiable visual loss in England and Wales during the year ending 31 March 2013. Eye. 2016;30(4):602-7.

4. Bourne RRA, Jonas JB, Bron AM, et al; Vision Loss Expert Group of the Global Burden of Disease Study. Prevalence and causes of vision loss in high-income countries and in Eastern and Central Europe in 2015: magnitude, temporal trends and projections. Br J Ophthalmol. 2018;102(5):575-585.

5. NG81. Glaucoma: diagnosis and management. Nov 2017. NICE. https://www.nice.org.uk/guidance/ng81

6. The management of angle closure glaucoma. Clinical Guideline. June 2022. Royal College of Ophthalmologists. https://www.rcophth.ac.uk/resources-listing/management-of-angle-closure-glaucoma-guideline/

7. Chua PY, Day AC, Lai KL, Hall N, Tan LL, Khan K, Lim LT, Foot B, Foster PJ, Azuara-Blanco A. The incidence of acute angle closure in Scotland: a prospective surveillance study. Br J Ophthalmol. 2018;102(4):539-543.

8. Foster PJ, Ng WS, Nolan WP, et al. Prevention of angle-closure glaucoma: balancing risk and benefit. Eye 2022;36:2229-2231.

9. Azuara-Blanco A, Banister K, Boachie C, et al. Automated imaging technologies for the diagnosis of glaucoma: a comparative diagnostic study for the evaluation of the diagnostic accuracy, performance as triage tests and cost-effectiveness (GATE study). Health Technology Assessment 2016;20:1-168.

10. Day AC, Baio G, Gazzard G, et al. The prevalence of primary angle closure glaucoma in European derived populations: a systematic review. Br J Ophthalmol. 2012;96(9):1162-1167.

For peer review only - http://bmjopen.bmj.com/site/about/guidelines.xhtml

11. Jindal A, Ctori I, Virgili G, Lucenteforte E, Lawrenson JG. Non-contact tests for identifying people at risk of primary angle closure glaucoma. Cochrane Database of Systematic Reviews 2020, Issue 5. Art. No.: CD0129.

12. Foster PJ, Devereux JG, Alsbirk PH, et al. Detection of gonioscopically occludable angles and primary angle closure glaucoma by estimation of limbal chamber depth in Asians: modified grading scheme. Br J Ophthalmol 2000;84:186–92.

13. Janssen MF, Pickard AS, Golicki D, et al. Measurement properties of the EQ-5D-5L compared to the EQ-5D-3L across eight patient groups: a multi-country study. Qual Life Res. 2013;22(7):1717-27.

14. Tun TA, Baskaran M, Tan SS, et al. Evaluation of the Anterior Segment Angle-to-Angle Scan of Cirrus High-Definition Optical Coherence Tomography and Comparison With Gonioscopy and With the Visante OCT. Invest Ophthalmol Vis Sci.2017;58(1):59-64.

15. Rigi M, Bell NP, Lee DA, et al. Agreement between Gonioscopic Examination and Swept Source Fourier Domain Anterior Segment Optical Coherence Tomography Imaging. J Ophthalmol. 2016;2016:1727039.

16. Dabasia PL, Edgar DF, Murdoch IE, Lawrenson JG. Noncontact Screening Methods for the Detection of Narrow Anterior Chamber Angles. Invest Ophthalmol Vis Sci. 2015;56(6):3929-35.

17. Genders TS1, Spronk S, Stijnen T, et al. Methods for calculating sensitivity and specificity of clustered data: a tutorial. Radiology. 2012;265(3):910-6.

18. Tang J, Liang Y, O'Neill C, Kee F, Jiang J, Congdon N. Cost-effectiveness and cost-utility of population-based glaucoma screening in China: a decision-analytic Markov model. Lancet Glob Health. 2019;7(7):e968-e978.

19. Husereau D, Drummond M, Augustovski F, et al.; CHEERS 2022 ISPOR Good Research Practices Task Force. Consolidated Health Economic Evaluation Reporting Standards 2022 (CHEERS 2022) Statement: Updated Reporting Guidance for Health Economic Evaluations. Value Health. 2022;25(1):3-9.

For peer review only - http://bmjopen.bmj.com/site/about/guidelines.xhtml

Appendix 1. Schedule of Assessments

Patients will be required to attend one study visit (Baseline/Visit 1). At this visit, eligibility will be confirmed, consent obtained and all study procedures will be completed.

Baseline/Visit 1

Step 1

Confirmation of eligibility by an ophthalmologist.

Written patient informed consent obtained to participate in the ACE study.

Step 2

Patients will undergo the following tests:

- AS-OCT, by an ophthalmic photographer/imaging technician
- Limbal anterior chamber depth (LACD), by a hospital optometrist
- Gonioscopy, by an ophthalmologist glaucoma expert

Step 3

Patients will complete the EQ-5D-5L questionnaire.

Step 4

The patients' information obtained during the standard care pathway will be recorded in the CRF including details on:

- Demographics and postal code
- Relevant medical and ophthalmic history
- Best corrected visual Acuity
- Refractive error

For peer review only
For peer review only - http://bmjopen.bmj.com/site/about/guidelines.xhtml

- Intraocular pressure
- Details on the presence/absence of glaucomatous damage
- If there is glaucomatous damage the MD value of the visual field test and reliability (percentage of false positives) will be recorded, if available.
- Any significant ocular co-morbidity

All ocular data will be obtained for both eyes separately.

Step 5

AS-OCT scans will be anonymised and transferred to QUB reading centre where they will be uploaded to an electronic website developed for the ACE study. The reading centre will then create folders and make these anonymised images accessible to the optometrists, ophthalmic photographers/imaging technicians and ophthalmologists.

Step 6

AS-OCT images will be interpreted by optometrists, ophthalmic photographers/technicians and ophthalmologists, masked to the reference standard (gonioscopy by ophthalmologists).

Optometrists, ophthalmic photographers/imaging technicians and ophthalmologists reading the images will not evaluate images of patients from their own institution to assure masking (see below). Once the images are read, the optometrists, ophthalmic photographers/imaging technicians and ophthalmologists will determine:

- whether there is angle closure or not
- whether they are unsure and reasons (e.g., poor image quality)

For peer review only - http://bmjopen.bmj.com/site/about/guidelines.xhtml

The optometrists, ophthalmic photographers/imaging technicians and ophthalmologists will record this information in the appropriate CRF.

For peer review only

For peer review only - http://bmjopen.bmj.com/site/about/guidelines.xhtml

Figure 1: ACE Study Flowchart

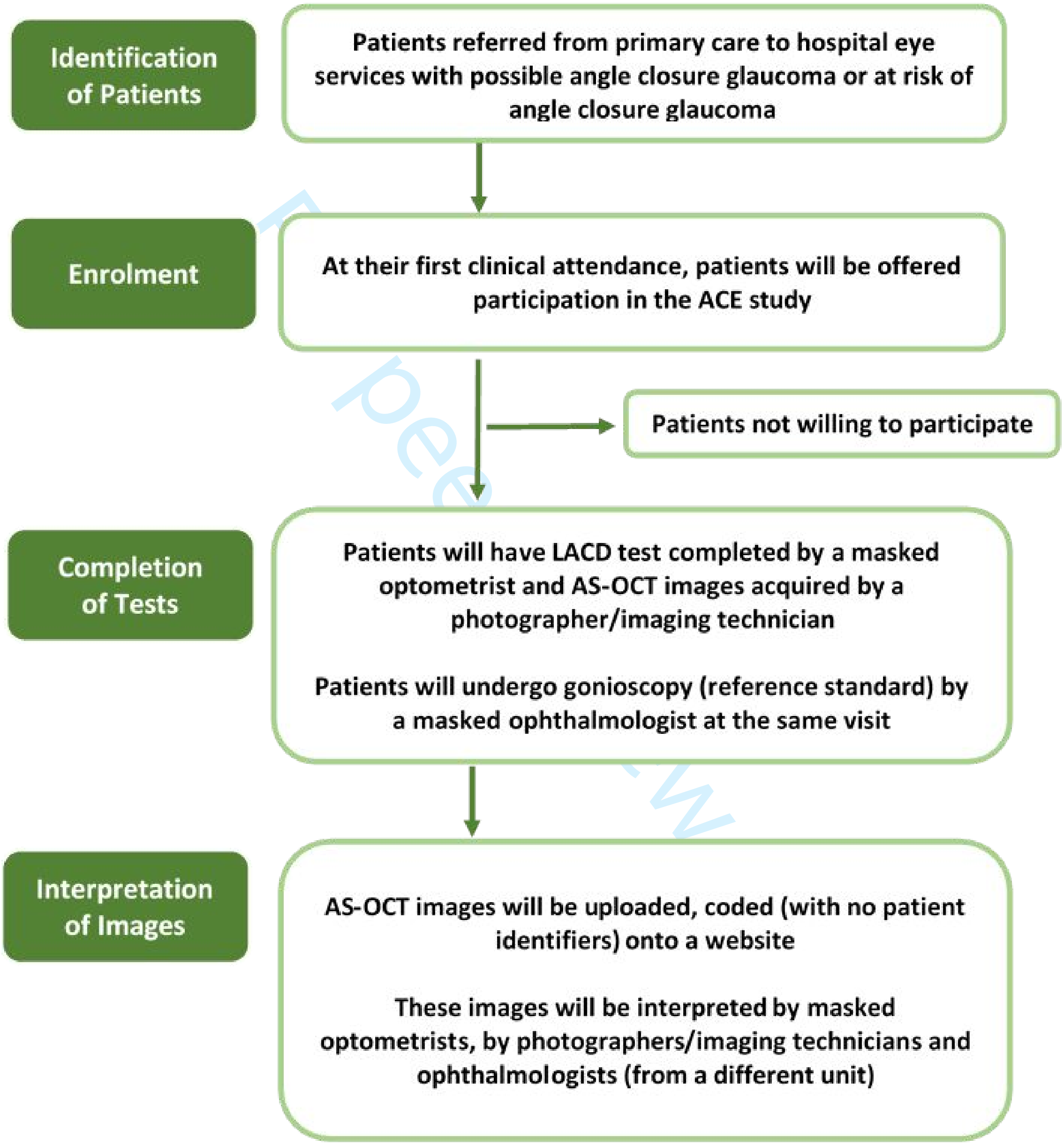

For peer review only - http://bmjopen.bmj.com/site/about/guidelines.xhtml

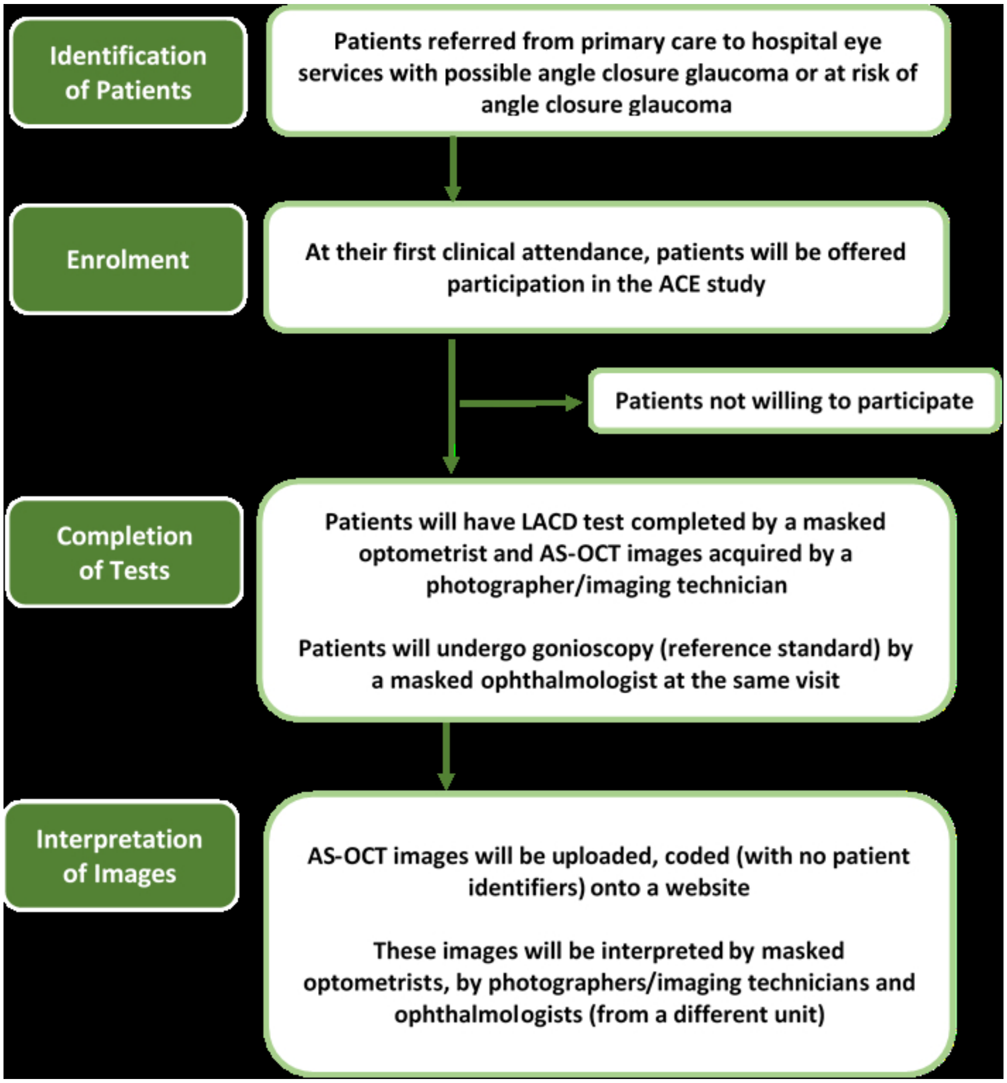

Figure 1. Participants flowchart

382x410mm (47 x 47 DPI)

Reporting checklist for protocol of a clinical trial.

Based on the SPIRIT guidelines.

Instructions to authors

Complete this checklist by entering the page numbers from your manuscript where readers will find each of the items listed below.

Your article may not currently address all the items on the checklist. Please modify your text to include the missing information. If you are certain that an item does not apply, please write "n/a" and provide a short explanation.

Upload your completed checklist as an extra file when you submit to a journal.

In your methods section, say that you used the SPIRITreporting guidelines, and cite them as:

Chan A-W, Tetzlaff JM, Gøtzsche PC, Altman DG, Mann H, Berlin J, Dickersin K, Hróbjartsson A, Schulz KF, Parulekar WR, Krleža-Jerić K, Laupacis A, Moher D. SPIRIT 2013 Explanation and Elaboration: Guidance for protocols of clinical trials. BMJ. 2013;346:e7586

		Reporting Item	Page Number
**Administrative information**			
Title	#1	Descriptive title identifying the study design, population, interventions, and, if applicable, trial acronym	1

For peer review only

Trial registration	#2a	Trial identifier and registry name. If not yet registered, name of intended registry	2
Trial registration: data set	#2b	All items from the World Health Organization Trial Registration Data Set	n/a
Protocol version	#3	Date and version identifier	5
Funding	#4	Sources and types of financial, material, and other support	14
Roles and responsibilities: contributorship	#5a	Names, affiliations, and roles of protocol contributors	15
Roles and responsibilities: sponsor contact information	#5b	Name and contact information for the trial sponsor	14
Roles and responsibilities: sponsor and funder	#5c	Role of study sponsor and funders, if any, in study design; collection, management, analysis, and interpretation of data; writing of the report; and the decision to submit the report for publication, including whether they will have ultimate authority over any of these activities	15
Roles and responsibilities: committees	#5d	Composition, roles, and responsibilities of the coordinating centre, steering committee, endpoint adjudication committee, data management team, and	8

For peer review only

		other individuals or groups overseeing the trial, if applicable (see Item 21a for data monitoring committee)	
**Introduction**			
Background and rationale	#6a	Description of research question and justification for undertaking the trial, including summary of relevant studies (published and unpublished) examining benefits and harms for each intervention	4-5
Background and rationale: choice of comparators	#6b	Explanation for choice of comparators	5
Objectives	#7	Specific objectives or hypotheses	5
Trial design	#8	Description of trial design including type of trial (eg, parallel group, crossover, factorial, single group), allocation ratio, and framework (eg, superiority, equivalence, non-inferiority, exploratory)	5
**Methods: Participants, interventions, and outcomes**			
Study setting	#9	Description of study settings (eg, community clinic, academic hospital) and list of countries where data will be collected. Reference to where list of study sites can be obtained	5

For peer review only

Eligibility criteria	#10	Inclusion and exclusion criteria for participants. If applicable, eligibility criteria for study centres and individuals who will perform the interventions (eg, surgeons, psychotherapists)	6
Interventions: description	#11a	Interventions for each group with sufficient detail to allow replication, including how and when they will be administered	6
Interventions: modifications	#11b	Criteria for discontinuing or modifying allocated interventions for a given trial participant (eg, drug dose change in response to harms, participant request, or7 improving / worsening disease)	n/a
Interventions: adherance	#11c	Strategies to improve adherence to intervention protocols, and any procedures for monitoring adherence (eg, drug tablet return; laboratory tests)	7
Interventions: concomitant care	#11d	Relevant concomitant care and interventions that are permitted or prohibited during the trial	n/a
Outcomes	#12	Primary, secondary, and other outcomes, including the specific measurement variable (eg, systolic blood pressure), analysis metric (eg, change from baseline, final value, time to event), method of aggregation (eg, median, proportion), and time point for each outcome. Explanation of the clinical relevance of chosen efficacy and harm outcomes is strongly recommended	6

For peer review only

Participant timeline	#13	Time schedule of enrolment, interventions (including any run-ins and washouts), assessments, and visits for participants. A schematic diagram is highly recommended (see Figure)	Figure 1
Sample size	#14	Estimated number of participants needed to achieve study objectives and how it was determined, including clinical and statistical assumptions supporting any sample size calculations	10
Recruitment	#15	Strategies for achieving adequate participant enrolment to reach target sample size	6
**Methods: Assignment of interventions (for controlled trials)**			
Allocation: sequence generation	#16a	Method of generating the allocation sequence (eg, computer-generated random numbers), and list of any factors for stratification. To reduce predictability of a random sequence, details of any planned restriction (eg, blocking) should be provided in a separate document that is unavailable to those who enrol participants or assign interventions	n/a
Allocation concealment mechanism	#16b	Mechanism of implementing the allocation sequence (eg, central telephone; sequentially numbered, opaque,	n/a

For peer review only

		sealed envelopes), describing any steps to conceal the sequence until interventions are assigned	
Allocation: implementation	#16c	Who will generate the allocation sequence, who will enrol participants, and who will assign participants to interventions	n/a
Blinding (masking)	#17a	Who will be blinded after assignment to interventions (eg, trial participants, care providers, outcome assessors, data analysts), and how	6
Blinding (masking): emergency unblinding	#17b	If blinded, circumstances under which unblinding is permissible, and procedure for revealing a participant's allocated intervention during the trial	n/a
**Methods: Data collection, management, and analysis**			
Data collection plan	#18a	Plans for assessment and collection of outcome, baseline, and other trial data, including any related processes to promote data quality (eg, duplicate measurements, training of assessors) and a description of study instruments (eg, questionnaires, laboratory tests) along with their reliability and validity, if known. Reference to where data collection forms can be found, if not in the protocol	7

For peer review only

Data collection plan: retention	#18b	Plans to promote participant retention and complete follow-up, including list of any outcome data to be collected for participants who discontinue or deviate from intervention protocols	7
Data management	#19	Plans for data entry, coding, security, and storage, including any related processes to promote data quality (eg, double data entry; range checks for data values). Reference to where details of data management procedures can be found, if not in the protocol	7
Statistics: outcomes	#20a	Statistical methods for analysing primary and secondary outcomes. Reference to where other details of the statistical analysis plan can be found, if not in the protocol	11
Statistics: additional analyses	#20b	Methods for any additional analyses (eg, subgroup and adjusted analyses)	11
Statistics: analysis population and missing data	#20c	Definition of analysis population relating to protocol non-adherence (eg, as randomised analysis), and any statistical methods to handle missing data (eg, multiple imputation)	n/a

Methods: Monitoring

Data monitoring: formal committee	#21a	Composition of data monitoring committee (DMC); summary of its role and reporting structure; statement of whether it is independent from the sponsor and	9

For peer review only

		competing interests; and reference to where further details about its charter can be found, if not in the protocol. Alternatively, an explanation of why a DMC is not needed	
Data monitoring: interim analysis	#21b	Description of any interim analyses and stopping guidelines, including who will have access to these interim results and make the final decision to terminate the trial	n/a
Harms	#22	Plans for collecting, assessing, reporting, and managing solicited and spontaneously reported adverse events and other unintended effects of trial interventions or trial conduct	n/a
Auditing	#23	Frequency and procedures for auditing trial conduct, if any, and whether the process will be independent from investigators and the sponsor	8
**Ethics and dissemination**			
Research ethics approval	#24	Plans for seeking research ethics committee / institutional review board (REC / IRB) approval	14
Protocol amendments	#25	Plans for communicating important protocol modifications (eg, changes to eligibility criteria, outcomes, analyses) to relevant parties (eg, investigators, REC / IRBs, trial participants, trial registries, journals, regulators)	14

For peer review only

Consent or assent	#26a	Who will obtain informed consent or assent from potential trial participants or authorised surrogates, and how (see Item 32)	13
Consent or assent: ancillary studies	#26b	Additional consent provisions for collection and use of participant data and biological specimens in ancillary studies, if applicable	n/a
Confidentiality	#27	How personal information about potential and enrolled participants will be collected, shared, and maintained in order to protect confidentiality before, during, and after the trial	14
Declaration of interests	#28	Financial and other competing interests for principal investigators for the overall trial and each study site	1
Data access	#29	Statement of who will have access to the final trial dataset, and disclosure of contractual agreements that limit such access for investigators	15
Ancillary and post trial care	#30	Provisions, if any, for ancillary and post-trial care, and for compensation to those who suffer harm from trial participation	n/a
Dissemination policy: trial results	#31a	Plans for investigators and sponsor to communicate trial results to participants, healthcare professionals, the public, and other relevant groups (eg, via publication, reporting in results databases, or other data sharing arrangements), including any publication restrictions	14-15

For peer review only

Dissemination policy: authorship	#31b	Authorship eligibility guidelines and any intended use of professional writers	15
Dissemination policy: reproducible research	#31c	Plans, if any, for granting public access to the full protocol, participant-level dataset, and statistical code	15

Appendices

Informed consent materials	#32	Model consent form and other related documentation given to participants and authorised surrogates	appendix
Biological specimens	#33	Plans for collection, laboratory evaluation, and storage of biological specimens for genetic or molecular analysis in the current trial and for future use in ancillary studies, if applicable	n/a

None The SPIRIT Explanation and Elaboration paper is distributed under the terms of the Creative Commons Attribution License CC-BY-NC. This checklist can be completed online using https://www.goodreports.org/, a tool made by the EQUATOR Network in collaboration with Penelope.ai

For peer review only

Technologies for the Diagnosis of Angle Closure glaucoma (ACE). Protocol of a prospective, multi-centre, cross-sectional diagnostic study.

Journal:	*BMJ Open*
Manuscript ID	bmjopen-2023-073975.R1
Article Type:	Protocol
Date Submitted by the Author:	27-Jul-2023
Complete List of Authors:	Azuara-Blanco, Augusto ; Queen's University Belfast, Centre for Public Health<br>Lawrenson, John j.g.; City University of London<br>King, Anthony ; Nottingham University Hospitals NHS Trust, Department of Ophthalmology<br>Foster, Paul; University College London, Institute of Ophthalmology. Division of of Epidemiology<br>Virgili, Gianni ; Queen's University Belfast, Centre for Public Health<br>Guiney, Mary; Belfast Health and Social Care Trust, Northern Ireland Clinical Trials Unit<br>Nolan, Winnie; Moorfields Eye Hospital City Road Campus, Glaucoma Service<br>Jindal, Anish; Moorfields Eye Hospital City Road Campus<br>Sharma, Meenakshi; Queen's University Belfast, Centre for Public Health<br>O'Neill, Ciaran; Queen's University Belfast, Centre for Public Health<br>Cardwell, Christopher; Queen's University Belfast, School of Medicine, Dentistry and Biomedical Sciences<br>Clarke, Mike; Queen's University Belfast, Northern Ireland Methodology Hub
**Primary Subject Heading**:	Diagnostics
Secondary Subject Heading:	Ophthalmology, Evidence based practice, Health economics, Health services research
Keywords:	OPHTHALMOLOGY, Glaucoma < OPHTHALMOLOGY, Clinical Trial, Diagnostic Imaging

SCHOLARONE™
Manuscripts

Technologies for the Diagnosis of Angle Closure glaucoma (ACE). Protocol of a prospective, multi-centre, cross-sectional diagnostic study.

- Augusto Azuara-Blanco; Centre for Public Health, Queen's University Belfast
- John G Lawrenson; Department of Optometry and Visual Sciences, City University of London
- Anthony King; Department of Ophthalmology, Nottingham University Hospitals NHS Trust
- Paul Foster; Institute of Ophthalmology. Division of Epidemiology, University College London
- Gianni Virgili; Centre for Public Health, Queen's University Belfast
- Mary Guiney; Northern Ireland Clinical Trials Unit, Belfast Health and Social Care Trust
- Winnie Nolan; Glaucoma Service, Moorfields Eye Hospital City Road Campus
- Anish Jindal; Glaucoma Service, Moorfields Eye Hospital City Road Campus
- Meenakshi Sharma; Centre for Public Health, Queen's University Belfast
- Ciaran O'Neill; Centre for Public Health, Queen's University Belfast
- Christopher Cardwell; Centre for Public Health, Queen's University Belfast
- Mike Clarke; Centre for Public Health, Queen's University Belfast

Corresponding author: Augusto Azuara-Blanco (a.azuara-blanco@qub.ac.uk)

Conflicts of interest: none

For peer review only

Abstract

Introduction: Angle-closure is responsible for half of all glaucoma blindness globally. Patients with suspected glaucoma require assessment of the drainage angle by an experienced clinician. The goal of this study is to evaluate the diagnostic performance and cost-effectiveness of two non-contact tests, anterior segment OCT (AS-OCT) and limbal anterior chamber depth (LACD) for patients referred to hospital with suspected angle closure compared to gonioscopy by ophthalmologist.

Methods and Analysis. Study design: prospective, multi-centre, cross-sectional diagnostic accuracy study. Inclusion criteria: adults referred from community optometry to hospital with suspected angle closure.

Primary outcome: Sensitivity and specificity. Secondary outcomes: Positive/negative likelihood ratios, concordance, cost -effectiveness, proportion of patients requiring subsequent clinical assessment by ophthalmologist.

Sample size: 600 individuals who have been referred with suspected angle closure from primary care (community optometry). We will have a 95% probability of detecting the true sensitivity of either test to within ±3.5% based upon a sensitivity of 90%. The study would also have a 95% probability of detecting the true specificity of either test to within ±5%, assuming a specificity of 75%.

Ethics and Dissemination: Ethical Review Board approval was obtained. REC reference: 22/LO/0885. Our findings will be disseminated to those involved in eye care services. We will have a knowledge exchange event at the end of the study, publish via the Health Technology Assessment webpage and in specialist journals. The results will be presented at professional conferences and directly to patients via patient group meetings and the Glaucoma UK charity.

Registration: ISRCTN registry – 43107

For peer review only

For peer review only - http://bmjopen.bmj.com/site/about/guidelines.xhtml

Strengths and limitations of this study:

Strengths:

- prospective study design

- masked investigators interpreting tests and the reference standard

- adequate, large sample size

- evaluation of cost-effectiveness of the novel patient pathway

Limitations:

- imperfect, subjective reference standard

Keywords: angle-closure glaucoma; diagnosis; anterior-segment optical coherence tomography; limbal anterior chamber depth; gonioscopy

Word count: 3798

For peer review only

Introduction

Glaucoma is a chronic optic neuropathy and a leading cause of irreversible blindness.(1-4) In the UK, more than 500,000 people have glaucoma, and 4000 new patients are registered each year with sight loss because of glaucoma. Many more people have glaucoma not severe enough to be registered, but severe enough to reduce vision and quality of life (e.g., loss of their driving licence).(5)

Hospital eye services (HES) account for 8% of outpatient attendances to the NHS and glaucoma accounts for 25% of outpatient activity of hospital eye services, with more than 1 million visits per year in England alone due to glaucoma.(5)

There are two types of glaucoma, according to the appearance of the drainage angle of the eye: open angle glaucoma (OAG), the most common, and angle closure glaucoma (ACG). In ACG, which is the subject of this study, the drainage angle is blocked, leading to acute or chronic elevation of intraocular pressure (IOP) and damage of the optic nerve.

ACG is less common but more severe than OAG and its prevalence increases with age [Bourne, Tham]. Acute angle closure is an uncommon but serious ocular emergency.(6,7) In the early stage of the disease, angle closure can be treated with laser to reduce the risk of developing glaucoma and vision loss.(6,8)

ACG is responsible for approximately 1 in 6 glaucomas in the UK and a substantial proportion of glaucoma referrals to hospital eye services.(9,10)

A recent Cochrane review found sub-optimal quality evidence regarding the diagnostic performance of non-contact tests, AS-OCT and LACD.(11) Pooled data showed that LACD had high sensitivity and a possibly sufficient specificity for case finding and performed as well as AS-OCT but the authors highlighted “There is still a need for high-quality studies to evaluate the performance of non-contact tests for angle assessment.” Given the demands OAG presents the health service and the implications delays in diagnosis may have for patients, clearly it is important to assess the cost-effectiveness of potential improvements to

For peer review only - http://bmjopen.bmj.com/site/about/guidelines.xhtml

the diagnostic pathway. Our study will fill this gap and determine the potential of these tests in the diagnosis of angle closure.

Our hypothesis is that the non-contact tests for diagnosing angle closure will be accurate and will facilitate a safe and efficient pathway for patients with this condition. The goal of this study is to evaluate the diagnostic performance and cost-effectiveness of two non-contact tests, anterior segment OCT (AS-OCT) and limbal anterior chamber depth (LACD) for patients referred to hospital with suspected angle closure compared to standard practice (gonioscopy by ophthalmologist). This report describes the protocol version 1.0.

Methods and analysis:

Study Design: ACE is a prospective, cross-sectional, multi-centre, diagnostic accuracy study of people referred to hospital eye services with suspected angle closure. Study setting is secondary care, hospital eye services.

Sampling and data collection will be carried out prospectively. Consecutive eligible patients will be approached to take part in the study (Figure 1). Patients referred to hospital eye services with suspected angle closure will be approached and those who consent will undergo testing with the two non-contact technologies (index tests) on the same day. Patients will undergo AS-OCT (with any device available at the site) and the images of the temporal and nasal angle will be sent to a reading centre for interpretation. Interpretation of AS-OCT will use reference images and the investigator will determine if the angle is closed (i.e., contact between peripheral iris and trabecular meshwork at one or both quadrants), open or indeterminate. In addition the investigator will grade the angle opening (<10 degrees, 10-20 degrees or >degrees) using reference images. The LACD will be performed and graded by a hospital optometrist (masked to the AS-OCT and to the reference standard). The investigator will use reference images to determine if the angle is closed (i.e., contact between peripheral iris and cornea), open, or indeterminate. The investigator will also grade the angle opening according to a 7-point scale proposed by Foster et al, using reference images.(12) Patients will then receive the reference standard (gonioscopy),

For peer review only - http://bmjopen.bmj.com/site/about/guidelines.xhtml

provided by an expert who will be masked to the evaluated tests (LACD and AS-OCT). The expert will be an ophthalmologist with glaucoma expertise and will judge if the angle is open or closed. Masked ophthalmologists, optometrists and trained photographers/imaging technicians will interpret AS-OCTs images provided by the reading centre at a different time. Prior the study recruitment we will meet clinicians and investigators at each study site to review and agree on the reference standard and interpretation of tests.

Inclusion criteria: Adults (≥18 years) referred from community optometry to hospital eye services with suspected angle closure, with or without additional co-morbidity. Exclusion criteria: Unable to provide informed consent; unable to undergo a slit-lamp examination.

The primary outcome is sensitivity and specificity of the non-contact tests to detect angle-closure. Reference standard is gonioscopy by an ophthalmologist with glaucoma expertise. Secondary outcomes: Positive/negative likelihood ratios, concordance, cost-effectiveness, proportion of patients requiring subsequent clinical assessment by ophthalmologist, proportion of patients unable to undergo tests and of tests of inadequate quality.

Recruitment of study participants: Potential patients will be identified through referral letters from optometrists to hospital eye services. Potential patients with possible angle closure will be approached before they come for their routine clinical appointment, by phone or via an invitation letter.

Prior to the initiation of the study, training will be provided to the investigators on the interpretation and grading of the tests (AS-OCT, LACD, gonioscopy) via a web-based teaching module on AS-OCT images and LACD interpretation.

The optometrists performing the LACD test will masked to gonioscopy. Optometrists, ophthalmic photographers/imaging technicians and ophthalmologists interpreting patients' images will be masked to the reference standard.

For peer review only

Ophthalmologists performing the gonioscopic evaluation will also be masked to the optometrist LACD test result and to the AS-OCT test, including the findings/decisions made by the optometrists, ophthalmic photographers/imaging technicians and ophthalmologists (who will be reviewing the images at a later date). At the same visit participants will be asked to complete a health status questionnaire, EQ-5D-5L.(13) The schedule of assessments is described in Appendix 1.

Data collection and management: the Chief Investigator (CI) and NICTU will provide training to site staff on trial processes and procedures, including the completion of the case report form (CRF) and data collection. Monitoring during the trial will check the accuracy of entries on CRF's against the source documents, the adherence to the protocol, procedures and GCP, as outlined in the trial monitoring plan.

Quality control is implemented by the NICTU in the form of Standard Operating Procedures (SOPs), which are defined to encompass aspects of the clinical data management process, and to ensure standardisation and adherence to International Conference of Harmonisation Good Clinical Practice (ICH-GCP) guidelines and regulatory requirements.

Data validation will be implemented and discrepancy reports will be generated following data entry to identify discrepancies such as out of range, inconsistencies or protocol deviations based on data validation checks programmed in the clinical trial database.

To ensure accurate, complete and reliable data are collected, the NICTU will provide training to site staff either through investigator meetings or site initiation visits.

All data for an individual patient will be collected by the PI or designee and recorded in source documents and/or the CRF for the study. The EQ-5D-5L questionnaires will be completed by the patient. Patient identification on the CRF and questionnaires will be through their unique trial identifier, allocated at the time of recruitment.

Case report forms and questionnaires are to be submitted to the NICTU as per the CRF submission schedule.

For peer review only - http://bmjopen.bmj.com/site/about/guidelines.xhtml

In addition to the data specified in the sections above, the following information will be obtained and recorded in the appropriate CRF:

1) The time the patient spent with a photographer/imaging technician to complete the AS-OCT images, the time the patient spent with the optometrist to complete the LACD test, and time the patient spent with the ophthalmologist to complete the gonioscopic examination. This information will be obtained in a consecutive group of patients until saturation is reached.
2) The time required by the optometrist, the ophthalmic photographer/imaging technician and the ophthalmologist to interpret the images and to determine whether there is angle closure or not.
3) Scores obtained in the health related quality of life questionnaire (EQ-5D-5L) filled in by patients and collected at the study visit, which will provide utility data for different health states.
4) Resource use data will be collected to explore the costs of delivering the standard care pathway and the new proposed triage pathway and to find the key cost drivers.

Trial Management Arrangements: the CI will have overall responsibility for the conduct of the study. The NICTU will undertake trial management including all clinical trial applications (Ethics and Research Governance), site initiation and training, monitoring, and reports to ethics, Sponsor and Funder. The trial co-ordinator will be responsible on a day to day basis for overseeing and co-ordinating the work of the multi-disciplinary trial team, and will be the main contact between the trial team and other parties involved. Before the trial starts, site training will take place to ensure that all relevant essential documents and trial supplies are in place and that site staff are fully aware of the trial protocol and procedures. The NICTU will assist and facilitate in the setting up and co-ordination of the trial committees.

Trial Management Group (TMG): a TMG will be established and Chaired by the CI. The TMG will include representation from the NICTU and other investigators or collaborators who are involved in the study and provide trial specific expertise (e.g. trial statistician, health

For peer review only

economist). This group will have responsibility for the day to day operational management of the trial. Regular meetings of the TMG will be held to discuss and monitor progress. The discussions of the TMG will be formally minuted and a record kept in the Trial Master File. A TMG Charter will be drawn up to detail the terms of reference of the TMG, including roles and responsibilities of the members.

Trial Steering Committee (TSC): the conduct of the trial will be overseen by an independent TSC. Throughout the study, the TSC will take responsibility for monitoring and guiding overall progress, scientific standards, operational delivery and protecting the rights and safety of patients enrolled in the study. The TSC will include an independent statistician, a health economist, at least two independent clinicians and a patient representative. The CI will attend the TSC meetings. Representatives of the Sponsor, Funder and the NICTU may attend TSC meetings as observers and at the discretion of the Chair. The TSC Charter will outline the terms of reference of the TSC including roles and responsibilities, membership, organisation of meetings (including frequency), reporting, decision making and the relationship with the other trial committees.

Data Monitoring and Ethics Committee (DMEC): the main role of the DMEC will be to monitor the trial data and make recommendations to the TSC on whether there are any ethical or safety reasons why the trial should not continue. The DMEC will include a researcher, a statistician and a clinician. Attendance at DMEC meetings by non-members will be at the discretion of the Chair. The primary DMEC reporting line will be via the Chair to the TSC. The DMEC Charter will outline the terms of reference of the DMEC including roles and responsibilities, membership, organisation of meetings (including frequency), reporting, decision making and the relationship with the other trial committees.

Patient and Public Involvement (PPI): the PPI co-applicants will be supported to convene a PPI Group that will actively contribute and advise on all patient- and public-facing documentation, including promotional patient information leaflets, informed consent forms and plain language summaries. They will also be involved in dissemination of the study's

For peer review only - http://bmjopen.bmj.com/site/about/guidelines.xhtml

results to patients and the public. The PPI group will meet during the development phase, at the end of the internal pilot phase (when the progression criteria are being considered) and at the end of the study.

Protocol Compliance: a protocol deviation is defined as an incident which deviates from the normal expectation of a particular part of the study process. Any deviations from the protocol will be fully documented on the protocol deviation form in the CRF.

A serious breach is defined as a deviation from the study protocol or GCP which is likely to effect to a significant degree:

(a) the safety or physical or mental integrity of the patients in the trial; or

(b) the scientific value of the trial

The PI or designee is responsible for ensuring that serious breaches are reported directly to the NICTU within one working day of becoming aware of the breach, using the dedicated email address (clinicaltrials@nictu.hscni.net). The NICTU will notify the CI and Sponsor immediately to ensure adherence to the reporting requirements to REC where a serious breach has occurred. Protocol compliance will be monitored by the NICTU who will undertake site visits to ensure that the trial protocol is adhered to and that necessary paperwork (e.g. CRFs, patient consent) is being completed appropriately.

The trial has been registered with ISCTRN (ISRCTN registry – 43107).

Statistical considerations

Sample size: we will recruit 600 individuals who have been referred with suspected angle closure from primary care (community optometry). According to our feasibility work, we estimate that approximately half of these 600 individuals will have angle closure and that half will not have it. Angle closure is typically a bilateral disease, and, thus, the majority of individuals will have similar angle characteristics in both eyes but, in the rare case of an individual having angle closure in only one eye, they will be considered to have angle closure.

For peer review only

Previous studies have shown that using a cut off to capture 90% sensitivity corresponds to a specificity of around 75% with AS-OCT or with LACD.(14-16) Therefore the study will have a 95% probability of detecting the true sensitivity of either test to within ±3.5% (i.e. the confidence interval for the true sensitivity would be approximately 7 percentage points in width), based upon a sensitivity of 90%. The study would also have a 95% probability of detecting the true specificity of either test to within ±5% (i.e., the confidence interval for the true specificity would be 10% in width), assuming a true specificity of 75%. These sample size are conservative because they are based on using only one eye per person, while in practice, as described below, information from both eyes will be used.

Statistical analysis: sensitivity and specificity will be calculated using data from both eyes, confidence intervals will then be calculated using variance inflation factors to account for the lack of independence of each eye in the same person (using the svy function in STATA).(17) Primary analysis will be based upon a binary result, closed or not closed. A sensitivity analysis will be conducted calculating test performance measures using a multi-level logistic regression model, with eyes nested within person, and person as a random effect. We will extend this model to include site and operator of the various tests to explore the impact these have on the width of the confidence intervals for sensitivity and specificity.

Secondary analyses will be conducted investigating different degrees of angle closure (on LACD and AS-OCT). Initially, a cut-off will be selected to obtain a sensitivity of 90% and the resulting specificity will be determined. We will explore the diagnostic accuracy of combining both tests (LACD and AS-OCT), and of using different thresholds for a positive result. We will also compare the diagnostic accuracy of AS-OCT images interpreted by optometrists, photographers/imaging technicians and ophthalmologists.

Health economics analysis: we will evaluate LACD and AS-OCT as part of a triage test to diagnose angle closure in patients referred to hospital eye services with possible glaucoma. We propose to use a Markov model to assess the longer term costs and effects of alternate diagnostic pathways. We propose to run this over an expected lifetime horizon. We propose to adapt a model developed and published by members of the study team.(18) The model

For peer review only - http://bmjopen.bmj.com/site/about/guidelines.xhtml

comprises 6 states – normal vision, suspected glaucoma, glaucoma without blindness, glaucoma-related unilateral blindness, glaucoma-related bilateral blindness and dead. Accurate testing may delay progression, through earlier identification and treatment. Transition probabilities, costs and outcomes (beyond those observed in the trial) associated with the various states will be taken from the literature, supplemented where appropriate with expert opinion and uncertainty explored using sensitivity analyses.

Three tests will be used in the study: gonioscopy as reference standard; LACD; AS-OCT and concordant LACD/AS-OCT results as triage tests. Three comparison groups will be created based on data collected: (A) comparison of gonioscopy versus LACD only; (B) comparison of gonioscopy versus AS-OCT only; and (C) comparison of gonioscopy versus LACD and AS-OCT concordant responses. The proportion of accurate diagnoses across modalities using gonioscopy as reference standard will provide a measure of effect and the differences in these will provide a measure of the incremental effect.

An NHS healthcare perspective will be used to evaluate cost effectiveness. For the cost-utility analysis, EQ-5D-5L data will also be gathered at baseline and provide the "starting point" against which subsequent decrements in health-related quality of life (HRQOL) arising from deteriorating vision will be compared. The trajectory of HRQOL after "normal vision" will be based on estimates from a systematic review of the literature for the health states specified in the Markov model. As HRQOL will vary based on age and time spent in a given state, adjustments for these will be made to reflect changes associated with cohort aging over repeated cycles of the model up to termination (death) for all members.

A similar approach will be adopted with respect to costs. In both cases a survey of experts will be used to address gaps in estimates and one-way and probabilistic sensitivity analysis will be used to explore uncertainty in such estimates. It is anticipated that cycle length will be one year.

The alternative test arrangements will have differential costs associated with them – for example, gonioscopy for all referrals likely being more expensive than those triaged based on LACD only or LACD only being less expensive than those triaged on a combination of

For peer review only - http://bmjopen.bmj.com/site/about/guidelines.xhtml

LACD and AS-OCT. They may also have differential effects in terms of identification for further investigation and treatment. While we anticipate false negatives/positives will be identified quickly without adverse health effects, the model will allow us to examine differential costs over a hypothetical lifetime for the cohort as well as to explore scenarios in which delayed identification does result in adverse effects.

Incremental QALYs and costs will be estimated for cohorts across triage and gonioscopy tests and expressed in terms of cost per QALY gain. Uncertainty around the threshold willingness to pay for a QALY will be explored using cost-effectiveness acceptability curves. Costs and outcomes will be discounted at 3.5% per annum as per current NICE guidance (https://www.nice.org.uk/process/pmg6/chapter/assessing-cost-effectiveness). Sub-group analyses will examine potential difference in ICERs across groups differentiated by age at screening sex and ethnic group. Reporting of results will adhere to revised-CHEERS checklist and ISPOR modelling good practice.(19)

Ethics and dissemination

The study will be conducted in accordance with the ethical principles that have their origin in the Declaration of Helsinki. Eligible patients will only be included in the trial after written informed consent is obtained. Informed consent must be obtained prior to conducting any trial specific procedures and the process for obtaining informed consent must be documented in the patient's medical records (source documents will be reviewed at the time of on-site monitoring visits).

Informed Consent Forms (ICF) approved by the REC will be provided by the NICTU. The PI or designee is responsible for ensuring that informed consent for trial participation is given by each patient prior to any trial procedure being performed. This requires that the ICF be signed and personally dated by the patient prior to any trial procedures are undertaken. If no consent is given, a patient cannot be recruited into the trial. Two copies of the ICF must be signed and personally dated by the patient and the individual taking consent. A copy of the

For peer review only - http://bmjopen.bmj.com/site/about/guidelines.xhtml

signed ICF will be filed in the patient's medical records, whilst the originals will be retained by the patient and by the PI in the Investigator Site File (ISF).

Queen's University Belfast (QUB) will act as Sponsor for the study and the CI will take overall responsibility for the conduct of the study (https://www.qub.ac.uk/Research/Research-contacts/). Separate agreements will be put in place between the Sponsor and each organisation who will undertake Sponsor delegated duties in relation to the management of the study.

Funding: funding was obtained from the National Institute of Health Research (NIHR) Health Technology Assessment Programme (HTA). Project ID: HTA NIHR134593. The views expressed are those of the author(s) and not necessarily those of the NIHR or the Department of Health and Social Care.

The protocol has been approved by an Ethical Review Board, the London - City & East Research Ethics Committee: REC reference:22/LO/0885, IRAS project ID: 315388.

Changes to the protocol may require ethics committee approval or favourable opinion prior to implementation. The NICTU in collaboration with the sponsor will submit all protocol modifications to the REC for review in accordance with the governing regulations.

In order to maintain confidentiality, all CRFs, questionnaires, study reports and communication regarding the study will identify the patients by their assigned unique trial identifier only. Computers where information will be stored will be password protected. Patient confidentiality will be maintained at every stage and will not be made publicly available to the extent permitted by the applicable laws and regulations.

Regarding dissemination, it is anticipated that the study findings will be published in national and international peer review journals and these articles will be led by the CI. This will secure a searchable compendium of these publications and make the results readily accessible to the public and health care professionals. In addition, study findings may be presented at both national and international meetings and to appropriate patient groups.

A report containing the methodology and results of this diagnostic study will be published as a Health Technology Assessment monograph, freely accessible via the NIHR HTA webpage.

For peer review only - http://bmjopen.bmj.com/site/about/guidelines.xhtml

The Royal College of Ophthalmologist will be contacted once the study is completed to allow the trial's findings to be incorporated in future glaucoma guidelines.

Data Access

Following the publication of the primary and secondary outcomes, there may be scope to conduct additional analyses on the data collected. In such instances, formal requests for data will need to be made in writing to the CI who will discuss this with the TMG and obtain approval from the Sponsor. In the event of publications arising from such analyses, those responsible will need to provide the CI with a copy of any intended manuscript for approval prior to submission. Authorship will need to take the format of "[name] on behalf of the ACE Clinical Trial Group" or something similar, which will be agreed by the TMG.

Author contributions. AAB, JGL, AK, PF, GV, CO'N, CC and MC are grant applicants are were involved in the initial study design and the first draft of the protocol, specifically clinical issues (AAB, JGL, AK, PF), statistical analyses (GV, CC), health economic considerations (CO'N) and overall trial design (MC). AJ and WN provided additional clinical input and helped with the development of the clinical aspects of the current protocol. MS contributed to the health economic aspects of the protocol. MG contributed to the trial management aspects. All authors contributed to the final version of the protocol and approved this submission.

Acknowledgements

Tara Holland, Ruth McCoy and Glaucoma UK for PPI support and advice .

For peer review only - http://bmjopen.bmj.com/site/about/guidelines.xhtml

References

1. Tham YC, Li X, Wong TY, et al. Global prevalence of glaucoma and projections of glaucoma burden through 2040: a systematic review and meta-analysis. Ophthalmology. 2014; 121:2081-90.

2. Jonas JB, Aung T, Bourne RR, et al. Glaucoma. Lancet. 2017;390(10108):2183-2193.

3. Quartilho A, Simkiss P, Zekite A, Xing W, Wormald R, Bunce C. Leading causes of certifiable visual loss in England and Wales during the year ending 31 March 2013. Eye. 2016;30(4):602-7.

4. Bourne RRA, Jonas JB, Bron AM, et al; Vision Loss Expert Group of the Global Burden of Disease Study. Prevalence and causes of vision loss in high-income countries and in Eastern and Central Europe in 2015: magnitude, temporal trends and projections. Br J Ophthalmol. 2018;102(5):575-585.

5. NG81. Glaucoma: diagnosis and management. Nov 2017. NICE. https://www.nice.org.uk/guidance/ng81

6. The management of angle closure glaucoma. Clinical Guideline. June 2022. Royal College of Ophthalmologists. https://www.rcophth.ac.uk/resources-listing/management-of-angle-closure-glaucoma-guideline/

7. Chua PY, Day AC, Lai KL, Hall N, Tan LL, Khan K, Lim LT, Foot B, Foster PJ, Azuara-Blanco A. The incidence of acute angle closure in Scotland: a prospective surveillance study. Br J Ophthalmol. 2018;102(4):539-543.

8. Foster PJ, Ng WS, Nolan WP, et al. Prevention of angle-closure glaucoma: balancing risk and benefit. Eye 2022;36:2229-2231.

9. Azuara-Blanco A, Banister K, Boachie C, et al. Automated imaging technologies for the diagnosis of glaucoma: a comparative diagnostic study for the evaluation of the diagnostic accuracy, performance as triage tests and cost-effectiveness (GATE study). Health Technology Assessment 2016;20:1-168.

10. Day AC, Baio G, Gazzard G, et al. The prevalence of primary angle closure glaucoma in European derived populations: a systematic review. Br J Ophthalmol. 2012;96(9):1162-1167.

For peer review only - http://bmjopen.bmj.com/site/about/guidelines.xhtml

11. Jindal A, Ctori I, Virgili G, Lucenteforte E, Lawrenson JG. Non-contact tests for identifying people at risk of primary angle closure glaucoma. Cochrane Database of Systematic Reviews 2020, Issue 5. Art. No.: CD0129.

12. Foster PJ, Devereux JG, Alsbirk PH, et al. Detection of gonioscopically occludable angles and primary angle closure glaucoma by estimation of limbal chamber depth in Asians: modified grading scheme. Br J Ophthalmol 2000;84:186–92.

13. Janssen MF, Pickard AS, Golicki D, et al. Measurement properties of the EQ-5D-5L compared to the EQ-5D-3L across eight patient groups: a multi-country study. Qual Life Res. 2013;22(7):1717-27.

14. Tun TA, Baskaran M, Tan SS, et al. Evaluation of the Anterior Segment Angle-to-Angle Scan of Cirrus High-Definition Optical Coherence Tomography and Comparison With Gonioscopy and With the Visante OCT. Invest Ophthalmol Vis Sci.2017;58(1):59-64.

15. Rigi M, Bell NP, Lee DA, et al. Agreement between Gonioscopic Examination and Swept Source Fourier Domain Anterior Segment Optical Coherence Tomography Imaging. J Ophthalmol. 2016;2016:1727039.

16. Dabasia PL, Edgar DF, Murdoch IE, Lawrenson JG. Noncontact Screening Methods for the Detection of Narrow Anterior Chamber Angles. Invest Ophthalmol Vis Sci. 2015;56(6):3929-35.

17. Genders TS1, Spronk S, Stijnen T, et al. Methods for calculating sensitivity and specificity of clustered data: a tutorial. Radiology. 2012;265(3):910-6.

18. Tang J, Liang Y, O'Neill C, Kee F, Jiang J, Congdon N. Cost-effectiveness and cost-utility of population-based glaucoma screening in China: a decision-analytic Markov model. Lancet Glob Health. 2019;7(7):e968-e978.

19. Husereau D, Drummond M, Augustovski F, et al.; CHEERS 2022 ISPOR Good Research Practices Task Force. Consolidated Health Economic Evaluation Reporting Standards 2022 (CHEERS 2022) Statement: Updated Reporting Guidance for Health Economic Evaluations. Value Health. 2022;25(1):3-9.

For peer review only - http://bmjopen.bmj.com/site/about/guidelines.xhtml

Figure legend.

Figure 1. ACE study participant flowchart.

For peer review only

For peer review only - http://bmjopen.bmj.com/site/about/guidelines.xhtml

Appendix 1. Schedule of Assessments

Patients will be required to attend one study visit (Baseline/Visit 1). At this visit, eligibility will be confirmed, consent obtained and all study procedures will be completed.

Baseline/Visit 1

Step 1

Confirmation of eligibility by an ophthalmologist.

Written patient informed consent obtained to participate in the ACE study.

Step 2

Patients will undergo the following tests:

- AS-OCT, by an ophthalmic photographer/imaging technician
- Limbal anterior chamber depth (LACD), by a hospital optometrist
- Gonioscopy, by an ophthalmologist glaucoma expert

Step 3

Patients will complete the EQ-5D-5L questionnaire.

Step 4

The patients' information obtained during the standard care pathway will be recorded in the CRF including details on:

- Demographics and postal code
- Relevant medical and ophthalmic history
- Best corrected visual Acuity
- Refractive error

For peer review only - http://bmjopen.bmj.com/site/about/guidelines.xhtml

- Intraocular pressure
- Details on the presence/absence of glaucomatous damage
- If there is glaucomatous damage the MD value of the visual field test and reliability (percentage of false positives) will be recorded, if available.
- Any significant ocular co-morbidity

All ocular data will be obtained for both eyes separately.

Step 5

AS-OCT scans will be anonymised and transferred to QUB reading centre where they will be uploaded to an electronic website developed for the ACE study. The reading centre will then create folders and make these anonymised images accessible to the optometrists, ophthalmic photographers/imaging technicians and ophthalmologists.

Step 6

AS-OCT images will be interpreted by optometrists, ophthalmic photographers/technicians and ophthalmologists, masked to the reference standard (gonioscopy by ophthalmologists).

Optometrists, ophthalmic photographers/imaging technicians and ophthalmologists reading the images will not evaluate images of patients from their own institution to assure masking (see below). Once the images are read, the optometrists, ophthalmic photographers/imaging technicians and ophthalmologists will determine:

- whether there is angle closure or not
- whether they are unsure and reasons (e.g., poor image quality)

For peer review only

The optometrists, ophthalmic photographers/imaging technicians and ophthalmologists will record this information in the appropriate CRF.

For peer review only

For peer review only - http://bmjopen.bmj.com/site/about/guidelines.xhtml

For peer review only

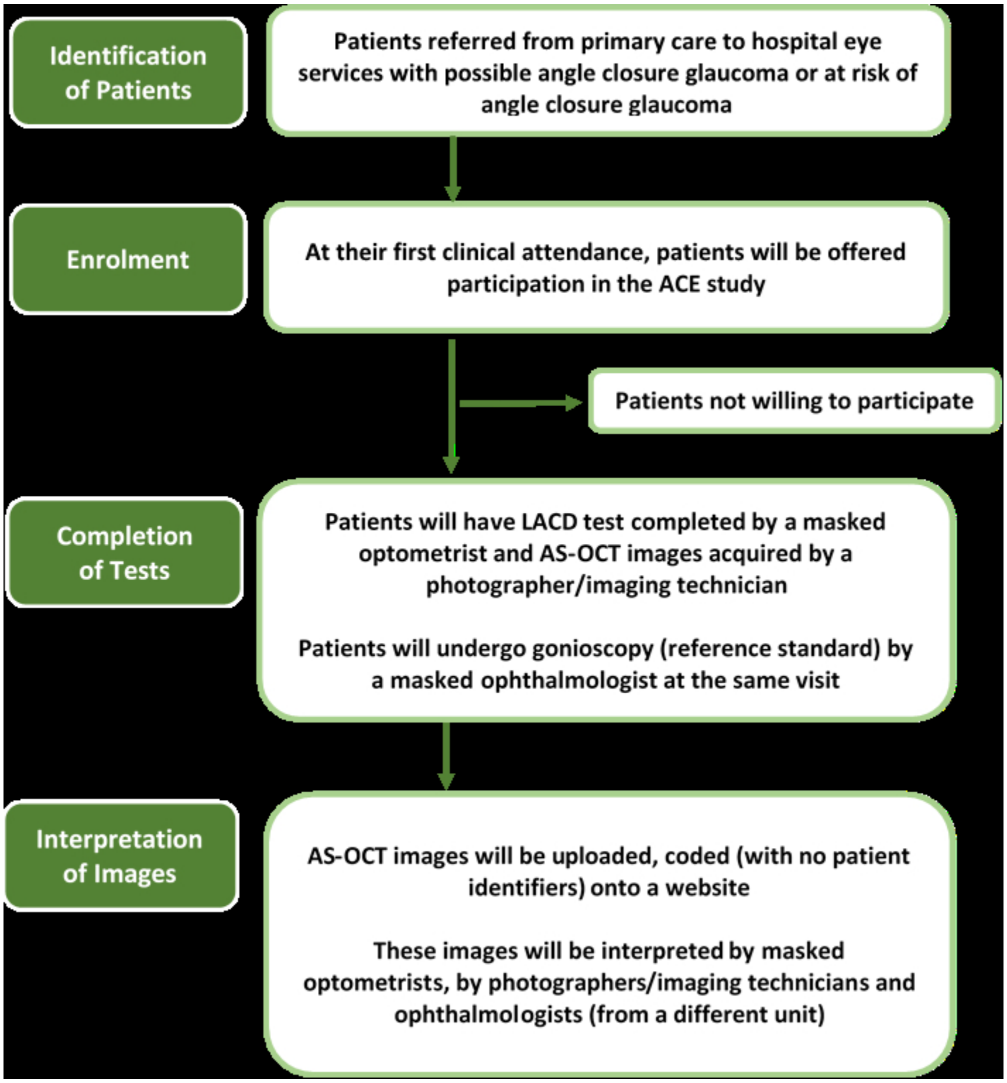

Figure 1. Participants flowchart

382x410mm (47 x 47 DPI)

Appendix 1. Schedule of Assessments

Patients will be required to attend one study visit (Baseline/Visit 1). At this visit, eligibility will be confirmed, consent obtained and all study procedures will be completed.

Baseline/Visit 1

Step 1

Confirmation of eligibility by an ophthalmologist.

Written patient informed consent obtained to participate in the ACE study.

Step 2

Patients will undergo the following tests:

- AS-OCT, by an ophthalmic photographer/imaging technician
- Limbal anterior chamber depth (LACD), by a hospital optometrist
- Gonioscopy, by an ophthalmologist glaucoma expert

Step 3

Patients will complete the EQ-5D-5L questionnaire.

Step 4

The patients' information obtained during the standard care pathway will be recorded in the CRF including details on:

- Demographics and postal code
- Relevant medical and ophthalmic history
- Best corrected visual Acuity
- Refractive error

For peer review only

- Intraocular pressure
- Details on the presence/absence of glaucomatous damage
- If there is glaucomatous damage the MD value of the visual field test and reliability (percentage of false positives) will be recorded, if available.
- Any significant ocular co-morbidity

All ocular data will be obtained for both eyes separately.

Step 5

AS-OCT scans will be anonymised and transferred to QUB reading centre where they will be uploaded to an electronic website developed for the ACE study. The reading centre will then create folders and make these anonymised images accessible to the optometrists, ophthalmic photographers/imaging technicians and ophthalmologists.

Step 6

AS-OCT images will be interpreted by optometrists, ophthalmic photographers/technicians and ophthalmologists, masked to the reference standard (gonioscopy by ophthalmologists).

Optometrists, ophthalmic photographers/imaging technicians and ophthalmologists reading the images will not evaluate images of patients from their own institution to assure masking (see below). Once the images are read, the optometrists, ophthalmic photographers/imaging technicians and ophthalmologists will determine:

- whether there is angle closure or not
- whether they are unsure and reasons (e.g., poor image quality)

For peer review only

The optometrists, ophthalmic photographers/imaging technicians and ophthalmologists will record this information in the appropriate CRF.

For peer review only

For peer review only

Reporting checklist for protocol of a clinical trial.

Based on the SPIRIT guidelines.

Instructions to authors

Complete this checklist by entering the page numbers from your manuscript where readers will find each of the items listed below.

Your article may not currently address all the items on the checklist. Please modify your text to include the missing information. If you are certain that an item does not apply, please write "n/a" and provide a short explanation.

Upload your completed checklist as an extra file when you submit to a journal.

In your methods section, say that you used the SPIRITreporting guidelines, and cite them as:

Chan A-W, Tetzlaff JM, Gøtzsche PC, Altman DG, Mann H, Berlin J, Dickersin K, Hróbjartsson A, Schulz KF, Parulekar WR, Krleža-Jerić K, Laupacis A, Moher D. SPIRIT 2013 Explanation and Elaboration: Guidance for protocols of clinical trials. BMJ. 2013;346:e7586

		Reporting Item	Page Number
**Administrative information**			
Title	#1	Descriptive title identifying the study design, population, interventions, and, if applicable, trial acronym	1

For peer review only

Trial registration	#2a	Trial identifier and registry name. If not yet registered, name of intended registry	2
Trial registration: data set	#2b	All items from the World Health Organization Trial Registration Data Set	n/a
Protocol version	#3	Date and version identifier	5
Funding	#4	Sources and types of financial, material, and other support	14
Roles and responsibilities: contributorship	#5a	Names, affiliations, and roles of protocol contributors	15
Roles and responsibilities: sponsor contact information	#5b	Name and contact information for the trial sponsor	14
Roles and responsibilities: sponsor and funder	#5c	Role of study sponsor and funders, if any, in study design; collection, management, analysis, and interpretation of data; writing of the report; and the decision to submit the report for publication, including whether they will have ultimate authority over any of these activities	15
Roles and responsibilities: committees	#5d	Composition, roles, and responsibilities of the coordinating centre, steering committee, endpoint adjudication committee, data management team, and	8

For peer review only

		other individuals or groups overseeing the trial, if applicable (see Item 21a for data monitoring committee)	
**Introduction**			
Background and rationale	#6a	Description of research question and justification for undertaking the trial, including summary of relevant studies (published and unpublished) examining benefits and harms for each intervention	4-5
Background and rationale: choice of comparators	#6b	Explanation for choice of comparators	5
Objectives	#7	Specific objectives or hypotheses	5
Trial design	#8	Description of trial design including type of trial (eg, parallel group, crossover, factorial, single group), allocation ratio, and framework (eg, superiority, equivalence, non-inferiority, exploratory)	5
**Methods: Participants, interventions, and outcomes**			
Study setting	#9	Description of study settings (eg, community clinic, academic hospital) and list of countries where data will be collected. Reference to where list of study sites can be obtained	5

For peer review only

Eligibility criteria	#10	Inclusion and exclusion criteria for participants. If applicable, eligibility criteria for study centres and individuals who will perform the interventions (eg, surgeons, psychotherapists)	6
Interventions: description	#11a	Interventions for each group with sufficient detail to allow replication, including how and when they will be administered	6
Interventions: modifications	#11b	Criteria for discontinuing or modifying allocated interventions for a given trial participant (eg, drug dose change in response to harms, participant request, or7 improving / worsening disease)	n/a
Interventions: adherance	#11c	Strategies to improve adherence to intervention protocols, and any procedures for monitoring adherence (eg, drug tablet return; laboratory tests)	7
Interventions: concomitant care	#11d	Relevant concomitant care and interventions that are permitted or prohibited during the trial	n/a
Outcomes	#12	Primary, secondary, and other outcomes, including the specific measurement variable (eg, systolic blood pressure), analysis metric (eg, change from baseline, final value, time to event), method of aggregation (eg, median, proportion), and time point for each outcome. Explanation of the clinical relevance of chosen efficacy and harm outcomes is strongly recommended	6

For peer review only

Participant timeline	#13	Time schedule of enrolment, interventions (including any run-ins and washouts), assessments, and visits for participants. A schematic diagram is highly recommended (see Figure)	Figure 1
Sample size	#14	Estimated number of participants needed to achieve study objectives and how it was determined, including clinical and statistical assumptions supporting any sample size calculations	10
Recruitment	#15	Strategies for achieving adequate participant enrolment to reach target sample size	6
**Methods: Assignment of interventions (for controlled trials)**			
Allocation: sequence generation	#16a	Method of generating the allocation sequence (eg, computer-generated random numbers), and list of any factors for stratification. To reduce predictability of a random sequence, details of any planned restriction (eg, blocking) should be provided in a separate document that is unavailable to those who enrol participants or assign interventions	n/a
Allocation concealment mechanism	#16b	Mechanism of implementing the allocation sequence (eg, central telephone; sequentially numbered, opaque,	n/a

For peer review only

		sealed envelopes), describing any steps to conceal the sequence until interventions are assigned	
Allocation: implementation	#16c	Who will generate the allocation sequence, who will enrol participants, and who will assign participants to interventions	n/a
Blinding (masking)	#17a	Who will be blinded after assignment to interventions (eg, trial participants, care providers, outcome assessors, data analysts), and how	6
Blinding (masking): emergency unblinding	#17b	If blinded, circumstances under which unblinding is permissible, and procedure for revealing a participant’s allocated intervention during the trial	n/a
**Methods: Data collection, management, and analysis**			
Data collection plan	#18a	Plans for assessment and collection of outcome, baseline, and other trial data, including any related processes to promote data quality (eg, duplicate measurements, training of assessors) and a description of study instruments (eg, questionnaires, laboratory tests) along with their reliability and validity, if known. Reference to where data collection forms can be found, if not in the protocol	7

For peer review only

Data collection plan: retention	#18b	Plans to promote participant retention and complete follow-up, including list of any outcome data to be collected for participants who discontinue or deviate from intervention protocols	7
Data management	#19	Plans for data entry, coding, security, and storage, including any related processes to promote data quality (eg, double data entry; range checks for data values). Reference to where details of data management procedures can be found, if not in the protocol	7
Statistics: outcomes	#20a	Statistical methods for analysing primary and secondary outcomes. Reference to where other details of the statistical analysis plan can be found, if not in the protocol	11
Statistics: additional analyses	#20b	Methods for any additional analyses (eg, subgroup and adjusted analyses)	11
Statistics: analysis population and missing data	#20c	Definition of analysis population relating to protocol non-adherence (eg, as randomised analysis), and any statistical methods to handle missing data (eg, multiple imputation)	n/a

Methods: Monitoring

Data monitoring: formal committee	#21a	Composition of data monitoring committee (DMC); summary of its role and reporting structure; statement of whether it is independent from the sponsor and	9

For peer review only

		competing interests; and reference to where further details about its charter can be found, if not in the protocol. Alternatively, an explanation of why a DMC is not needed	
Data monitoring: interim analysis	#21b	Description of any interim analyses and stopping guidelines, including who will have access to these interim results and make the final decision to terminate the trial	n/a
Harms	#22	Plans for collecting, assessing, reporting, and managing solicited and spontaneously reported adverse events and other unintended effects of trial interventions or trial conduct	n/a
Auditing	#23	Frequency and procedures for auditing trial conduct, if any, and whether the process will be independent from investigators and the sponsor	8
**Ethics and dissemination**			
Research ethics approval	#24	Plans for seeking research ethics committee / institutional review board (REC / IRB) approval	14
Protocol amendments	#25	Plans for communicating important protocol modifications (eg, changes to eligibility criteria, outcomes, analyses) to relevant parties (eg, investigators, REC / IRBs, trial participants, trial registries, journals, regulators)	14

For peer review only

Consent or assent	#26a	Who will obtain informed consent or assent from potential trial participants or authorised surrogates, and how (see Item 32)	13
Consent or assent: ancillary studies	#26b	Additional consent provisions for collection and use of participant data and biological specimens in ancillary studies, if applicable	n/a
Confidentiality	#27	How personal information about potential and enrolled participants will be collected, shared, and maintained in order to protect confidentiality before, during, and after the trial	14
Declaration of interests	#28	Financial and other competing interests for principal investigators for the overall trial and each study site	1
Data access	#29	Statement of who will have access to the final trial dataset, and disclosure of contractual agreements that limit such access for investigators	15
Ancillary and post trial care	#30	Provisions, if any, for ancillary and post-trial care, and for compensation to those who suffer harm from trial participation	n/a
Dissemination policy: trial results	#31a	Plans for investigators and sponsor to communicate trial results to participants, healthcare professionals, the public, and other relevant groups (eg, via publication, reporting in results databases, or other data sharing arrangements), including any publication restrictions	14-15

For peer review only

Dissemination policy: authorship	#31b	Authorship eligibility guidelines and any intended use of professional writers	15
Dissemination policy: reproducible research	#31c	Plans, if any, for granting public access to the full protocol, participant-level dataset, and statistical code	15
**Appendices**			
Informed consent materials	#32	Model consent form and other related documentation given to participants and authorised surrogates	appendix
Biological specimens	#33	Plans for collection, laboratory evaluation, and storage of biological specimens for genetic or molecular analysis in the current trial and for future use in ancillary studies, if applicable	n/a

None The SPIRIT Explanation and Elaboration paper is distributed under the terms of the Creative Commons Attribution License CC-BY-NC. This checklist can be completed online using https://www.goodreports.org/, a tool made by the EQUATOR Network in collaboration with Penelope.ai

For peer review only
