# Supplementary material for: Technologies for the diagnosis of angle closure glaucoma (ACE): protocol of a prospective, multicentre, cross-sectional diagnostic study
Source: BMJ Open. 2023 Oct 4;13(10):e073975. doi: 10.1136/bmjopen-2023-073975 (PMC10551982; doi:10.1136/bmjopen-2023-073975)
Supplement: Reviewer comments [file bmjopen-2023-073975.reviewer_comments.pdf]

PEER REVIEW HISTORY

BMJ Open publishes all reviews undertaken for accepted manuscripts. Reviewers are asked to complete a checklist review form (**http://bmjopen.bmj.com/site/about/resources/checklist.pdf**) and are provided with free text boxes to elaborate on their assessment. These free text comments are reproduced below.

ARTICLE DETAILS

**TITLE (PROVISIONAL)**	Technologies for the Diagnosis of Angle Closure glaucoma (ACE). Protocol of a prospective, multi-centre, cross-sectional diagnostic study.
**AUTHORS**	Azuara-Blanco, Augusto; Lawrenson, John j.g.; King, Anthony; Foster, Paul; Virgili, Gianni; Guiney, Mary; Nolan, Winnie; Jindal, Anish; Sharma, Meenakshi; O'Neill, Ciaran; Cardwell, Christopher; Clarke, Mike

This article was not externally reviewed at BMJ Open. Protocols that have been independently assessed prior to submission to BMJ Open are usually fast-tracked to publication on the grounds that further substantial changes will not be possible. This independent assessment will usually be external, independent review for both a substantial grant award from a non-commercial or government funder and ethics approval.
